# Advances in the detection technology of vegetable soil borne fungi and bacteria

**DOI:** 10.3389/fmicb.2024.1460729

**Published:** 2024-12-04

**Authors:** Lida Chen, Guiyun Lü, Songhan Yang, Binbin Gong, Yusong Lu, Xiaolei Wu, Jingrui Li, Hongbo Gao

**Affiliations:** Key Laboratory of North China Water-Saving Agriculture, Ministry of Agriculture and Rural Affairs, Hebei Key Laboratory of Vegetable Germplasm Innovation and Utilization, Collaborative Innovation Center of Vegetable Industry in Hebei, College of Horticulture, Hebei Agricultural University, Baoding, China

**Keywords:** vegetable, soil borne pathogens, detection techniques, primer information, physiological races

## Abstract

Soil borne diseases are one of the most serious diseases which often results the decline of vegetables quality and loss of production. Moreover, it is difficult for plants to exhibit disease symptoms in the early stages attributing to strong concealment of soil borne pathogens. Therefore, early detection of pathogens and their physiological races plays an important role in reducing the harm of pathogens associated with diseases of vegetable crops. The traditional diagnostic techniques relied on the time consuming and less accurate methods like disease symptom observation, microscopic diagnosis, and culture techniques etc. The development of molecular biology technology has brought revolutionary changes to the diagnosis of vegetable soil borne diseases, improving the accuracy and efficiency of diagnosis. This paper reviews the various molecular detection techniques for vegetable soil borne pathogens (PCR, nested-PCR, multiplex PCR, etc.) and their physiological races (host identification, DNA molecular markers, transposon detection, etc.), explains the advantages and disadvantages of each detection technique. Furthermore, the paper comprehensively introduces the application of molecular detection technology for soil borne pathogen detection in soil, plants, and seeds. Finally, we put forward important perspectives for the future development of rapid detection methods, aiming to promote rapid diagnosis of soil pathogenic microorganisms and provide guidance for the control of biological risks.

## 1 Introduction

Vegetables are among the essential foods in people’s daily diet and are also used in food, nutrition, healthcare, etc. (Willer et al., 2023). As per reports of the Food and Agriculture Organization (FAO), China is the first largest global producer of vegetables, with a planting area of 23.42 million·hm^2^ (FAO, 2023). However, with the continuous cultivation of vegetables for several years, soil borne pathogens have accumulated and cropping obstacles have become seriously increasing. The typical soil borne pathogens commonly found including fungi (*Fusarium* sp., *Rhizoctonia* sp., *Phytophthora* sp., etc) and bacteria (*Ralstonia* sp., *Pectobacterium* sp., *Clavibacter* sp., etc). The infected plants developed slowly, their leaves defoliated and wilted, their roots turned brown, with decayed cortical tissues that shriveled. Due to the long incubation period, complexity and rapid spread of soil borne pathogens, soil borne diseases cause huge economic losses to vegetables (Xie et al., 2024).

The early detection of vegetable soil borne pathogens is arguably the most challenging task, mainly due to unclear disease symptoms in the early stages, their complex and diverse physiological races, and limitations of different detection methods. Notably, physiological races of vegetable soil borne pathogens are particularly difficult to detect due to their high similarity. Traditional techniques for detecting soil-borne pathogens, such as the use of selective media, are of great value because they are relatively inexpensive and not technically demanding. Microscopic diagnosis is a fast technique to identify the spores of soil borne fungal pathogens in vegetables. Traditional detection methods such as symptom observation, microscopic diagnosis, pathogen plate culture technology, are time consuming, laborious, low sensitivity and not suitable for the rapid as well as early diagnosis of soil borne diseases. New technologies, such as molecular biology detection technology, provide better means for the diagnosis as well as study of vegetable diseases causing pathogens and physiological race with high reliability, precision and accuracy.

This review focused on the various molecular detection techniques for vegetable soil borne pathogens (conventional PCR, nested-PCR, multiplex PCR, RT-qPCR, PMA-qPCR, LAMP, RPA/CRISPR-Cas12a, genomics) and their physiological races (host identification, conventional PCR, DNA molecular markers, transposon, pathogenicity-related genes, genomics), also explained the advantages and disadvantages of each detection technique. Moreover, the new technology, both currently in use and under development were also described, for diagnosing soil borne diseases of vegetables, with an emphasis on the application of different detection techniques in different tissues (soil, plants, and seeds). This review is the comprehensive summary about the progress and application of recent molecular detection techniques for vegetable soil borne diseases. Moreover, providing the references for the further development and application of biology detection technology in the detection of pathogenic microorganisms for disease prevention and control.

## 2 Molecular detection techniques for soil borne pathogens in vegetables

### 2.1 Conventional PCR based detection technology

With the rapid development of modern biotechnology, detection methods based on molecular biology have widely adopted. At present, *16S rDNA*, *ITS* sequences and other genes (*RBP1*, *RBP2*, *TEF-1α*, *gyrB*) are mainly used as templates to design specific primers for PCR amplification. The use of PCR based detection technology has been widely reported for vegetable soil borne pathogens (*Fusarium oxysporum*, *R. solani*, *Verticillium dahliae*, *Phytophthora capsici*, etc.) (Table 1). The advantages of PCR technology include the ability to detect single pathogens and non-culturable pathogens in complex mixed soil/plant samples (Shneyder et al., 2022). Although the detection speed of conventional PCR is fast at low cost and sensitivity, thus, it can only be used for qualitative analysis. Moreover, the distribution of pathogens in soil is uneven, which can lead to false negatives in conventional PCR detection.

**Table 1:** Primer information for common PCR detection of soil borne pathogens.

Disease	Pathogen	Primer sequence (5′-3′)	Target gene	Fragment size (bp)	References
Root rot	*Fusarium* sp.	F8-1:GCTTCTCCCGAGTCCCA	EF-1α	187	Chen et al. (2019)
		F8-2:GCTCAGCGGCTTCCTAT			
		EF1:ATGGGTAAGGARGACAAGAC		639–683	O'Donnell et al. (2010)
		EF2:GGARGTACCAGTSATCATG			
		Fa:CAYAARGARTCYATGATGGGWC	RPB1	1,607	
		G2R:GTCATYTGDGTDGCDGGYTCDCC			
		5f2:GGGGWGAYCAGAAGAAGGC	RPB2	1,700–1,742	
		7cr:CCCATRGCTTGYTTRCCCAT			
	*Fusarium oxysporum*	FOR1-F:TTCCACAGCCAAGTGTGATCTTCAC	EF-1α	610	Kim et al. (2017)
		FOR1-R:TTACTCGCGCTTTATCCCAGTAATAGC			
Sheath blight	*Rhizoctonia solani*	F: CTCAAACAGGCATGCTC	28S	300	Matsumoto (2002)
		R: AGGCAATAGGTTATTGGACC			
Gummy stem blight	*Stagonosporopsis* spp.	DBF1: TCGAATGGCTCAGAGAAGGT	RGI	559	Murolo et al. (2022)
		DBR1: AAGTCCACGTCAGACCCATC			
Sclerotinia rot	*Sclerotinia* sp.	Pg1R: TCTTGCAGCAGTCGAGAAGA	Pg	495	Oliveira et al. (2010)
		Pg1F: GTGTTGTGTCCGAGGGAGTT			
Verticillium wilt	*Verticillium dahliae*	VActF: TAATTCACAATGGAGGGTAGG	Actin	530	Gharbi et al. (2015)
		VActR: GTAAGGATACCACGCTTGG			
Anthracnose	*Colletotrichum* spp.	F: AACCCTTTGTGAACRTACCTA	ITS	460	Martinez-Culebras et al. (2003)
		R: TTACTACGCAAAGGAGGC			
Phytophthora blight	*Phytophthora capsici*	Pc1F: GTATAGCAGAGGTTTAGTGAA	Ypt1	364	Lan et al. (2013)
		Pc1R: ACTGAAGTTCTGCGTGCGTT			
Late blight	*Phytophthora infestans*	Yph1F: CGACCATKGGTGTGGACTTT		203	Khan et al. (2017)
		Yph2R: ACGTTCTCMCAGGCGTATCT			
Damping-off	*Pythium aphanidermatum*	AsAPH2B: GCGCGTTGTTCACAATAAATTGC	ITS	163	Asano et al. (2010)
		AsPyF:CTGTTCTTTCCTTGAGGTG			
Bacterial wilt	*Ralstonia solanacearum*	RS-1-F:ACTAACGAAGCAGAGATGCATTA	18S rRNA	716	Pastrik et al. (2002)
		RS-3-R:TTCACGGCAAGATCGCTC			
Bacterial soft rot	*Pectobacterium carotovorum* subsp*.brasiliense*	BR1f:GCGTGCCGGGTTTATGACCT		374	Duarte et al. (2004)
		L1r:CARGGCATCCACCGT			
Bacterial canker	*Clavibacter michiganensis* sub sp. *michiganensis*	Fan1:GCATGTGCACCTCTCCTCTGTA		146	Wu et al. (2007)
		Fan2:CCCCACAAGGAGGCGTACTA			

### 2.2 Nested-PCR based detection technology

The nested PCR technique uses two pairs of PCR primers to amplify the complete fragment (Koentjoro et al., 2023). The main advantage is that the results of the second amplification can change the erroneous fragments produced by the first amplification. Nested PCR techniques have been developed to detect latent infections caused by fungi (Mutasa et al., 1996; Grote et al., 2002; Mudiyanselage et al., 2021), bacteria (Liop et al., 2000) and viruses (Nair and Manimekalai, 2021) in host plants. A nested PCR assay developed for rapid detection of *F. oxysporum* f. sp. *lactucae* in lettuce seeds permitted the detection of the pathogen in seed lots with an infestation rate as low as 0.1% (Mbofung and Pryor, 2010). Klemsdal and Elen developed a nested PCR detection technique for *Fusarium culmorum* with a detection limit of 5–50 fg of purified target DNA, and its sensitivity is 100 times higher than that of conventional PCR (Klemsdal and Elen, 2006). Li et al. (2014) established a nested PCR detection system for *Phytophthora* with detection limits of 100 fg of genomic DNA per 25-μL reaction. Qin et al. (2011) established a quick and accurate technique to detect infection of rape oil seed by *S. sclerotiorum* via a nested PCR technique, which can detect 50 fg of genomic DNA in approximately 6 h. Jesús et al. (2002) designed specific primers based on the DNA (RAPD) marker sequence with a band size of 1958 bp and established a nested PCR technique for the detection of non-defoliating (ND) *V. dahliae*. However, detection technology based on nested PCR still has some shortcomings. First, this technique is time consuming, as it requires two PCRs followed by confirmation of the positive result by agarose gel electrophoresis. Second, in open environments where multiple samples are processed, nested PCR is more prone to contamination (Cordova et al., 2014). The above shortcomings often lead to false positives, thus reducing the efficiency of molecular diagnosis of pathogens associated with vegetable diseases (Youssef et al., 2017).

### 2.3 Multiplex PCR based detection technology

Multiplex PCR (M-PCR) is a variant of PCR in which two or more target sequences are simultaneously amplified in the same reaction, combining the advantages of conventional PCR and nested PCR (Israa et al., 2023). M-PCR is more practical in diagnostics and research in the vegetable crops thus, saves time and cost, as many pathogens often infect the same vegetable (Panno et al., 2014). Notably, construction of the M-PCR system requires evaluation of the compatibility of specific primers of multiple pathogens. Ozdemir (2005) used dual PCR to detect tomato canker (Cmm) and scab (Xav). Gao et al. (2010) established an M-PCR detection technique for the pathogens *Cladosporium cucumerinum*, *F. oxysporum* and *Mycosphaerella melonis* in infected plant tissues. The M-PCR detection technology established by Quinterovásquez et al. (2010) can simultaneously detect *Clavibacter* and *Fusarium* in tomato which has high application value. Umesha and Avinash (2015) developed a dual PCR technology that can be used to detect bacterial wilt and scab in vegetables. Gao et al. (2016) established an M-PCR detection technology to successfully measure the levels of *Corynespora cassiicola, Colletotrichum orbiculare*, and *Pseudomonas syringae* pv. *lachrymans*. Kang et al. (2018) designed specific primers for quadruple PCR detection of tomato soil borne pathogens. It is rapid and stable technique, including the detection of *Pseudomonas syringae*, *C. michiganensis* subsp. *michiganensis*, *R. solanacearum*, and *Xanthomonas campestris* (Kang et al., 2018). Liu et al. (2019) established M-PCR technology for simultaneous detection of *P. aphanidermatum*, *F. oxysporum* and *V. dahliae* in vegetable fields, with improved efficiency and reduction in the time to detect each pathogen. Moreover, the three soil borne pathogens *R. solanacearum*, *V. dahliae* and *Sclerotium rolfsii* were identified in eggplant, using this M-PCR technology with the detection rate (Zou et al., 2023). The disadvantage is that multiple primer pairs, templates, etc. are prone to non-specific amplification in the same reaction, leading to false positive detection results. Other obvious limitations are if the length difference of amplified fragments is limited by the resolution of agarose gel electrophoresis, it may affect the detection sensitivity. It was unable to ascertain the pathogenicity and infectivity of the organism detected using M-PCR technology. Furthermore, it was unable to infer data on microbial cell integrity, which has an impact on epidemiological assessment. Therefore, in designing primers, it is necessary to choose a consistent PCR amplification system, especially the annealing temperature. Only in this way can the efficiency of soil borne pathogens detection be improved.

### 2.4 RT–qPCR based detection technology

Real-time quantitative PCR (RT–qPCR) is well developed detection technology. This technique uses fluorescence signals to monitor the amplification products of each cycle in the DNA replication process in real time *in-vitro* conditions. This technique allowing the quantitative and qualitative analysis of DNA templates, thus, has become the gold standard for vegetable disease diagnosis compared with conventional PCR technology (Chen et al., 2023). In particular, RT–qPCR can quantitatively detect non-culturable pathogens in vegetable tissues and pathogens that cannot be extracted from host tissue (Mirmajlessi et al., 2015; Abou-Jawdah et al., 2019). The use of RT–qPCR detection technology has been widely reported in research on soil borne vegetable diseases (Table 2). Vojvodic et al. (2019) designed specific primers and developed an RT–qPCR technique for *R. solani* that was 100 times more sensitive than the conventional PCR technique. Chen et al. (2019) designed *Fusarium*-specific primers for the *TEF* gene and constructed an RT–qPCR detection system with the sensitivity 10,000 times higher than that of conventional PCR. Yang et al. (2022) designed specific primers and established an RT–qPCR technique for the detection of *Colletotrichum* spp. in strawberry with the detection limit of 10–10^5^ copies. Cheng et al. (2018) established an RT–qPCR detection system for *P. capsici* using the YM2F/YM2R primers, with a sensitivity of 10^−1^ pg.·μL^−1^, 100 times more than that of conventional PCR. The advantages of RT–qPCR are as follows: (i) speed: compared with conventional PCR, the main advantage of RT–qPCR is that the whole RT–qPCR runs can be performed in 1 to 2 h without complicated steps; (ii) sensitivity: RT–qPCR is 10 ~ 10^4^ times more sensitive than conventional PCR; (iii) specificity: evaluation of specificity can be done by melting curve analysis during operation; (iv) quantification: compared with conventional PCR, fluorescence PCR can be used to quantitatively measure the levels of vegetable pathogens through comparison with a standard curve. RT–qPCR technique is its inability to differentiate between live and dead cells of pathogens but identify their presence. Although this technology is efficient and applicable, there are limitations in the quantitative detection of soil borne pathogens. It is used mainly for research in the field of soil borne disease management. With the recognition of RT–qPCR detection technology by farmers, the demand for commercial detection is likely to increase.

**Table 2:** Primer information for common RT-qPCR detection of soil borne pathogens.

Disease	Pathogen	Primer sequence (5′-3′)	Target gene	Fragment size (bp)	References
Root rot	*Fusarium* sp.	F8-1:GCTTCTCCCGAGTCCCA	EF-1α	187	Chen et al. (2019)
		F8-2:GCTCAGCGGCTTCCTAT			
	*F. oxysporum*	18F:TAATGCTCGTAAGTCAGGTCAGGTCAGGTTCA		172	Zhong et al. (2022)
		18R:AGTTGGAGTCAGCGATTCAT			
	*Aphanomyces cochlioides*	AcF:TCCGGGCTAGCCGAAGGTT	rRNA	96	Almquist et al. (2016)
		AcR:ACAAGCAATCATTTCTGATGCTAGATAG			
Sheath blight	*Rhizoctonia solani*	AG-F:CACCTTTTGCTCTTTTTTTAATCCA	ITS	150	Vojvodic et al. (2019)
		AG-R:ATAAATTGGGTTTATATTAGAGTTGAGTAGACA			
Gummy stem blight	*Stagonosporopsis* spp.	DBF1:TCGAATGGCTCAGAGAAGGT	RGI	208	Murolo et al. (2022)
		DBR1:AAGTCCACGTCAGACCCATC			
Sclerotinia rot	*Sclerotinia* sp.	Scl SF:CTCAAATCTCCGAAAGTT	β-tubulin	237	Li et al. (2011)
		Scl AF:TGCAGACGGGTAATATG			
Verticillium wilt	*Verticillium dahliae*	VertBt-F:AACAACAGTCCGATGGATAATTC		115	Duressa et al. (2011)
		VertBt-R:GTACCGGGCTCGAGATCG			
Anthracnose	*Colletotrichum* spp.	cuti-F:AGAACCAGATCAAGGGCGTCGTG	Cutinase	222	Tavernier and Coenye (2015)
		cuti-R:GCGTCCGCAATGTCGCAGTA			
Phytophthora blight	*Phytophthora capsici*	YM2F:ATTCCTCCTGATAGATAG	Actin	245	Nocker et al. (2006)
		YM2R:CCCTCATCACAGAATGC			
Late blight	*Phytophthora infestans*	qPCR F:CATCGGTGTTGACTTTGTG	Ypt1	203	Khan et al. (2017)
		qPCRR:TGAGCAATGTAATGGCAATC			
Bacterial wilt	*Ralstonia solanacearum*	OLI-1:GGGGGTAGCTTGCTACCTGCC	16S rRNA	288	Ramesh et al. (2011)
		Y2: CCCACTGCTGCCTCCCTAGGAGT			
Bacterial fruit blotch	*Acidovorax citrulli*	Acf3: CCTCCACCAACCAATACGCT		550	Zhao et al. (2009)
		Aacr2: TCGTCATTACTGAATTTCAACA			
Bacterial canker	*Clavibacter michiganensis* sub sp. *michiganensis*	Cmm141F: CAGGCGTCCGTCGGTGAGGTGGTC	pat-1	141	Cho et al. (2012)
		Cmm141R: GCGGGAGAGCGGTGCGGGAATG			

### 2.5 PMA-qPCR based detection technology

PMA (Propidium Monoazide) is a special membrane impermeable dye so that can penetrate damaged cell membranes to emit fluorescent signals without direct impact on intact cells (Foteini et al., 2023). Following a specific duration of light reaction, PMA, after entering dead/ damaged cells, combines with their DNA. It causes the loss of fluorescence signals during bacterial DNA amplification and ignores the level of dead cells. The process of constructing a PMA-qPCR detection system for vegetable diseases includes screening the concentration of PMA and illumination time. PMA inhibit the PCR amplification of dead-cell DNA reducing the overestimation of cell count caused by dead-cell DNA in qPCR detection (Tavernier and Coenye, 2015). Since the first description by Nocker et al. (2006), PMA has been applied to a wide variety of microorganisms, including bacteria, viruses and fungi (Table 3). Chen L. et al. (2022) designed the specific primers F8-1/F8-2 based on the translation elongation factor (*TEF*) gene, screened PMA concentration (50 mmol·L^−1^) and illumination time (15 min). With this they established a PMA-qPCR technique to amplify and quantify living cells of *Fusarium* in soil. Xie X. W. et al. (2022) designed PMA-qPCR primers based on the *SdhB* sequence of the *Corynespora* blight pathogen, which effectively detect *C. cassiicola* in soil. Compared with its use to study vegetable-infecting, the application of PMA-qPCR detection technology in fungal research is relatively rare compared with studies on bacterial pathogens. This technology differentiate the dead and live cells of pathogens hence it is highly applicable in field disease control and drug screening. However, PMA is expensive and only suitable for professional laboratory personnel, not for field operation.

**Table 3:** Primer information for common PMA-qPCR detection of soil borne pathogens.

Disease	Primer sequence (5′-3′)	PMA concentration	Light time	Target gene	Fragment size (bp)	References
Root rot (*Fusarium* sp.)	F8-1:GCTTCTCCCGAGTCCCA	50 mmol·L^−1^	15 min	EF-1α	187	Chen L. et al. (2022)
	F8-2:GCTCAGCGGCTTCCTAT					
Early blight (*Alternaria* spp.)	Alt4:CTTTTGCGTACTTCTTGTTTCC	65 μmol·L^−1^	10 min	ITSs	240	Crespo-Sempere et al. (2013)
	Alt5:CAGGCATGCCCTTTGGATAC					
Corynespora blight (*Corynespora cassiicola*)	CC-F3:CAGGAAATCCTCGCCAAGCAG	80 μmol·L^−1^	10 min	SdhB	109	Xie X. et al. (2022)
	CC-R3:CGCCAGTGATACGGTTGAACGG					
Clubroot (*Plasmodiophora brassicae*)	PBF3:TCTTGCGTGTCGCTGTATTC	4 μmol·L^−1^	10 min	16S rDNA	~150	Li et al. (2022)
	PBR3:ATAGGTTGGGGTAACTTGGC					
Bacterial speck (*Pseudomonas syringae*)	Pst3F:GCTGCGGATGGCAAGTC	10 μmol·L^−1^	10 min	HrpZ	161	Chai et al. (2020)
	Pst3R:CCGACACCCGAACCAGAAC					
Bacterial wilt (*Ralstonia solanacearum*)	RS72F:ATGGATAAAGGGTTCGTGGTG	3 ng·μL^−1^	5 min	16S rDNA	241	Cao et al. (2015b)
	RS312R:CAGGCTCAGCGAGATTGC					
Bacterial fruit blotch (*Acidovorax citrulli*)	FP:CTGATAATCCTCGGCTCAACAA	3 μg·mL^−1^	5 min	ArgA	121	Tian et al. (2016)
	RP:TGAGCGCATTTCTGACGAG					
Cucumber angular leaf spot (*Pseudomonas syringae* pv. *lachrymans*)	Pslgap1-F:TCGGCGACGCAATCAAT	60 μmol·L^−1^	10 min	Gap1	162	Meng et al. (2016)
	Pslgap1-R:GGTGGTTTCACGCTTCAGG					
Bacterial canker (*Clavibacter michiganensis* sub sp. *michiganensis*)	Spm4f:TCAGGCGTCTGTTCTGGC	5 μmol·L^−1^	30 min	ITS	~150	Luo et al. (2008)
	Spm2r:CCCACCACCATCCACAAC					
Black spot (*Pseudomonas syringae*)	PM1:GCGAAGCGACAQCAACAGTG	10 μg·mL^−1^	10 min	FliC	~150	Yu et al. (2021)
	PM3:CGAGTCGATAGCGGCAAC					

### 2.6 LAMP based detection technology

LAMP (loop-mediated isothermal amplification), an isothermal nucleic acid amplification platform devised by Notomi, has emerged as a popular tool for phytoplasma molecular detection (Khat et al., 2024). The basic principle of this technique involves the design of four specific primers for six different regions on the target sequence, including a pair of internal primers (FIP and BIP), a pair of external primers (F3 and B3) and a pair of ring primers (LF and LB). The LAMP reaction can be simply carried out under isothermal conditions by using BstDNA polymerase with high displacement activity. LAMP technology has been widely used in the detection of soil borne pathogens in vegetables, such as bacteria (Gieroń et al., 2023), viruses (Supakitthanakorn et al., 2022), and oomycetes (Htun et al., 2020), because of its applicability in open field operations. In addition, there have also been widespread reports of the use of LAMP technology to detect soil borne pathogenic fungi in vegetables (Zeng et al., 2017; Achari et al., 2023; Xie X. W. et al., 2022). Peng et al. (2013) tested 8 artificially inoculated samples and 85 field soil samples with the LAMP technique, and the detection limit for *F. oxysporum* was 10^3^ spores. Huang W. et al. (2016) established LAMP technology for the detection of *R. solanacearum* in vegetables, and its sensitivity was 10 times higher than that of conventional PCR. Yao et al. (2016) constructed a LAMP detection system for *Didymella bryoniae*; its sensitivity was 1,000 times that of conventional PCR, and the detection limit was 0.1 fg·μL^−1^. Almasi (2019) used LAMP to detect the DNA of *F. oxysporum*, and the detection efficiency was 100 times greater than that of conventional PCR. For *Colletotrichum* species, LAMP detection exhibits accuracy and strong sensitivity, for the detection of pathogen DNA at 100 pg.·μL^−1^ (Liu Y. et al., 2021). Lu et al. (2015) designed and screened specific primers based on the *ITS* sequence of *R. solani* for establishing the LAMP detection system which allowed successful as well as rapid diagnosis for this species. The advantages of LAMP are as follows: (1) high amplification efficiency, 10–1,000 times higher than that of conventional PCR; (2) high speed, the whole reaction can be completed within 30–60 min; (3) strong specificity, the four specific primers for LAMP are for six conserved sites in the target gene sequence, and DNA amplification cannot be performed if any site does not match; (4) simple operation and (5) low cost. The disadvantage of LAMP is that it can only be used for qualitative analysis, not for quantitative detection. In addition, LAMP is prone to producing false positives due to its open operation, which affects the results.

### 2.7 RPA/CRISPR-Cas12a based detection technology

The clustered regularly inter spaced palindromic repeats/CRISPR-associated proteins system technology is currently an emerging nucleic acid detection technology. The principle is that RPA rapidly amplify the nucleic acid fragment to be detected at 37°C to achieve the minimum detection limit of CRISPR/Cas12a (Kim et al., 2023). After activation of the RuvC cleavage site of Cas12a, it will exert its non-specific nuclease activity (Zetsche et al., 2015; Chen et al., 2018). Cas12a can release fluorescent signals released by Cas12a captured by qPCR equipment, and the presence of nucleic acid fragments can be observed with the naked eye under ultraviolet/blue light (Ding et al., 2020; Wang et al., 2020). Currently, The RPA-CRISPR/Cas12a detection system has been used in the research of mycoplasma (Wang et al., 2019), COVID-19 (Ding et al., 2020), transgenic crops (Liu H. et al., 2021), rice diseases (Kang et al., 2021), etc., but there is no relevant report on the detection of vegetable soil borne diseases. Kuang et al. (2022) established a rapid detection method of RPA/CRISPR-Cas12a for specifically detecting black stem fungus by RPA reaction for 30 min under constant temperature 37°C and CRISPR-Cas12a reaction for 20 min. Lei et al. (2022) designed specific primers based on the *Ypt1* gene of *Phytophthora syringae* to established rapid detection methods using fluorescence and lateral flow chromatography strips. In this method gene was amplified at 37°C for 40 min and could specifically detect *P. syringae* with a sensitivity of 133 fg, which is equivalent to fluorescence quantitative PCR. Wei et al. (2024) established a rapid detection system for *F. pseudograminearum* using RPA/CRISPR-Cas12a, which can detect the target pathogen within 40 min under constant temperature conditions of 37°C and sensitivity can reach 10^−3^ pg.·μL^−1^. The detection results can be intuitively read through the color reaction of nucleic acid test strips. This system has the advantages of specificity, sensitivity, and speed (Wei et al., 2024). It is also simple operation, fast and flexible to operation, high throughput and automated, and does not require complex temperature control equipment. The test strip detection does not require fluorescence equipment such as excitation light sources, making it very suitable for developing on-site rapid detection platforms. However, the cost of RPA/CRISPR-Cas12a reagents is higher than PCR. Hence in the future, the cost can be reduced through innovation and optimization conditions, to have a broader application.

### 2.8 Genomics based detection technology

With the advent of high-throughput sequencing technology, the detection of soil borne pathogens in vegetables has advanced greatly. High-throughput second-generation sequencing and single-molecule long-read third-generation sequencing improved the accuracy of bacterial detection. Also this technique identify a variety of specific microbial populations, such as unknown bacteria, viruses and viroids. This technique does not require the design of primers for specific sequences of microorganisms or plate culture of microorganisms (Zhang X. et al., 2023; Zhang Y. et al., 2023), which is important because only approximately 10% of bacteria are culturable (Pace, 1997). Next-generation sequencing (NGS, for which various platforms exist, such as Solexa, 454 Roche, Illumina and Ion Torrent), pyrosequencing and metagenomics have been widely used in research on soil borne vegetable diseases (Hopkins et al., 2013). At present, the use of sequencing technology to identify unknown pathogens costs $850 and takes approximately 2 weeks or longer (Olmos et al., 2013). Yuan et al. (2020) detected high levels of *F. oxysporum*, *Gibberella*, *Bacillaceae*, and *Xanthomonadaceae* in diseased soil and *Streptomyces*, *Bradyrhizobiaceae*, *Comamonadaceae*, and *Mortierella* in healthy soil using high-throughput sequencing technology. Previous reports have detected the oomycetes and fungi *P. ultimum*, *P. irregulare*, *P. aphanidermatum*, *P. nicotianae*, *P. capsici*, *P. cinnamomi*, *R. solani*, and *F. oxysporum* in soil via genomics technology (Jambhulkar et al., 2015; Huang C. H. et al., 2016). Genomic technology can be used to detect unknown soil bacteria, which is not suitable for common disease diagnosis. Metagenomics sequencing is the sequencing of all DNA in the environment. The cost of genome sequencing is relatively high, and the computational resources required for subsequent data analysis are also relatively high. With further research and over time, the cost of this technology will gradually decrease. In contrast, amplicon technology has become the main means of environmental microbiome research due to its low cost advantage. Amplicon sequencing mainly targets ribosomal RNA genes and functional genes. The former amplifies molecular markers such as bacteria, archaea, fungi, and *ITS* sequences, while the latter amplifies specific functional genes in microorganisms (such as those involved in carbon and nitrogen cycling).

## 3 Molecular detection techniques for physiological races of soil borne pathogens in vegetables

### 3.1 Host based identification technology

Each strain of pathogen normally infects one or a few host species, and the host-specific form called forma specialis which is further divided into physiological races depending on their cultivar specificity. The traditional biological identification techniques for specialized forms of pathogens (e.g., *F. oxysporum*, *P. capsici*, *R. solanacearum*, *P. brassicae*) mainly rely on the pathogenicity of the pathogen on different species, while the identification of physiological races mainly relies on the pathogenicity on different varieties of the same host, which is time-consuming and labor-intensive (Martyn and Netzer, 1991; Geng et al., 2010; Ji et al., 2007; Williams, 1966; Buczack et al., 1975; Jones et al., 1982). There are some differences between routine pathogen identification and physiological race identification in vegetables. The former only requires pathogenicity testing on plants of the same genus, while the latter requires pathogenicity testing on different cultivars of same hosts, which is time-consuming and labor-intensive, and easily affected by different environmental conditions.

### 3.2 Conventional PCR based detection technology

Molecular biology identification techniques have the advantages of speed, high sensitivity, and high accuracy and thus have been applied in the identification of most physiological races of pathogenic bacteria (Rui et al., 2022; Fu et al., 2020). The *ITS* has been proposed as the barcode for fungal species identification. The *TEF* and the DNA-directed RNA polymerase II with their subumits via., largest subunit (*RPBI*) and second largest subunit (*RPB2*) are phylogenetically informative loci in *Fusarium* allowing for species identification (O'Donnell et al., 2015). The *TEF* locus is also informative at the intraspecific level and can be combined with others, such as the ribosomal intergenic spacer, to reveal the complex genetic diversity within *F. oxysporum* (Lecomte et al., 2016; Ortu et al., 2018). Generally, using *ITS*, *TEF*, *β-tubulin*, *Actin*, and *RPB* genes to design specific primers can effectively distinguish between genera or species of pathogens but cannot distinguish physiological races (Chang et al., 2018). To compensate for the above shortcomings, four pairs of specific primers, uni, sp13, sp23, and sprl, were used to amplify the DNA of the tomato wilt pathogen, which can effectively identify *Fol* 1, *Fol* 2, *Fol* 3, and FORL of *F. oxysporum* (Hirano and Arie, 2006).

### 3.3 DNA based molecular markers based detection technology

At present, molecular detection techniques based on specific primers have been successfully applied to the identification of many physiological races of pathogens (Lin et al., 2008; Cabanás et al., 2011; Aruga et al., 2012; Manzanares-dauleux et al., 2000) (Table 4). Cao et al. (2015a) screened a pair of primers, RS72F/RS312R, from a subtractive gene library of *R. solanacearum* and used qPCR technology to specifically amplify its physiological race 5. Zhang et al. (2015) utilized the nonhousekeeping genes reported by NCBI to screen and obtain Cr811, which can specifically identify race 5 of *P. brassicae*. In 2018, Zheng et al. (2018) screened molecular marker genes that could identify several physiological races of *P. brassicae*. The molecular markers PBRA_007750 and PBRA_009348 used for distinguishing P11 from P4, P7, and P9; PBRA_009348 and Novel342 used for distinguishing P9 from P4, P7, and P11; and PBRA_008439 and Novel342 could represent P4. Yi et al. (2020) screened two molecular marker genes, *PBRA*, based on previous transcriptome sequencing data_000030, and Novel00510, which can specifically identify the dominant physiological race 4 of *P. brassicae*. The polygalacturonase gene (*pgx*) is conserved, with high species specificity, has been widely used for the detection of physiological races of *Fusarium* (Lievens et al., 2009a). Ye et al. (2022) designed KASP-SNP primers based on the *pgx4* gene locus of *F. oxysporum* and for the first time developed KASP-SNP molecular markers for physiological races of *F. oxysporum*. The KASP-SNP technique used to detect *Fol* 1, *Fol* 2, *Fol* 3, and FORL rapidly and accurately. In the early stages of vegetable disease, the following specific primers can be used to identify the pathogen DNA, thus an early disease prevention and control plan can be formulated. The reading of KASP-SNP test data is completely automated, while the routine PCR test needs to be analyzed by agarose gel electrophoresis. The reading of test results are subjective, and errors are prone to occur in the judgment based on the clarity of the target strip. However, the above mentioned molecular marker techniques have drawbacks, such as requiring a large amount of DNA, poor repeatability, and complex operation.

**Table 4:** Primer information for physiological races of vegetable soil borne diseases.

Disease	Marker name	Primer sequence (5′-3′)	Fragment size (bp)
Root rot (*Fusarium* sp.)	Routine PCR	uni-F:ATCATCTTGTGCCAACTTCAG	670
		uni-R:GTTTGTGATCTTTGAGTTGCCA	
		sprl-F:GATGGTGGAACGGTATGACC	947
		sprl-R:CCATCACACAAGAACACAGGA	
		sp13-F:GTCAGTCCATTGGCTCTCTC	445
		sp13-R:TCCTTGACACCATCACAGAG	
		sp23-F:CCTCTTGTCTTTGTCTCACGA	518
		sp23-R:GCAACAGGTCGTGGGGAAAA	
		Fo-1F:CTGCCCGCTGGGAACAAGCT	1873
		Fo-3R:CTTAACCGTTGAACTTTCTAAC	
		ITS1:TCCGTAGGTGAACCTGCGG	544 ~ 568
		ITS4:TCCTCCGCTTATTGATATGC	
	FORL_KASP	FORL-FAM-f:GAAGGTGACCAAGTTCATGCTATGGTGGAACGGTATGACC	
		FORL-HEX-h:GAAGGTCGGAGTCAACGGATTATGGTGGAACGGTATGACT	
		FORL-c:AAGAATCTCCTTGCCGGCAAACTCTGCATA	
	FOLrace1_KASP	FOLrace1-FAM-f:GAAGGTGACCAAGTTCATGCTCCTTGAACGAGATGTCCTTGGCTAG	
		FOLrace1-HEX-h:GAAGGTCGGAGTCAACGGATTCCTTGAACGAGATGTCCTTGGCTAC	
		FOLrace1-c:GTCGTCACCTGTAAGGAACCCT	
	FOLrace2_KASP	FOLrace2-FAM-f:GAAGGTGACCAAGTTCATGCTGTCCTTGAAGTGAACTCCC	
		FOLrace2-HEX-h:GAAGGTCGGAGTCAACGGATTGTCCTTGAAGTGAACTCCT	
		FOLrace2-c:TCCGACCTATTCTGTTCTATGCT	
	FOLrace3_KASP	FOLrace3-FAM-f:GAAGGTGACCAAGTTCATGCTTTGTGTTAGTGCTACTAGTGCGGCA	
		FOLrace3-HEX-h:GAAGGTCGGAGTCAACGGATTTTGTGTTAGTGCTACTAGTGCGGCG	
		FOLrace3-c:TAACCTTGAAAGGGCTCGCAGAAGCCTGAG	
Bacterial wilt (*Ralstonia solanacearum*)	lpx C	RS72F:ATGGATAAAGGGTTCGTGGTG	241
		RS312R:CAGGCTCAGCGAGATTGC	
Club root (*Plasmodiophora brassicae*)	Cr811	ActF:GGGACATCACCGACTACCTG	492
		ActR:ACTGCTCCGAGTTGGACATC	
	PBRA	007750-1-F:CTTCGTGCTGACCGATTCCT	638
		007750-1-R:ATAATGCTCTGCGTCAGCCA	
		009348-1-F:CACTGCTATCGTCTCCCTGG	509
		009348-1-R:CCTGCAATGTTTCGCTGCAA	
		008439-1-F:TCGGCGACCTGAGCGAGAA	651
		008439-1-R:TCAACATGCGCATAGTAC	
		l342-1-F:TCCTCTTGAACCGACACTGC	249
		l342-1-R:CTTCTCTCGCACTAGCCAGG	

### 3.4 Transposon based detection technology

Transposons are ubiquitous in all organisms and pathogens (Lievens et al., 2008). Based on the genomic flanking regions, Pasquali et al. (2004) constructed a molecular identification technique for *F. oxysporum* physiological races using a special insertion fragment (Mg5/Mg6) of the *Fot* 1 transposon. Pasquali et al. (2007) indicated that inter-retrotranspos on sequence-characterized amplified regions (IR-SCAR) was used to develop a specific set of PCR primers (Ha3F/Ha3R) utilized for differentiating *F. oxysporum* race 1 from other *F. oxysporum* isolates. López-Berges et al. (2009) demonstrated that the artificially modified Impala transposon has a decisive effect on the toxicity of *F. oxysporum* on tomato by inserting it. Research has shown that the *Fot* 1 transposon can serve as a target sequence resource library for identifying specific physiological races (1, 2, 8) of *F. oxysporum*, while the Impala transposon can be used for identifying physiological race 4. Currently, the majority of their genomes have not been annotated for soil borne pathogens, and there is relatively little research on using transposon technology to identify physiological races (Table 5). The problem encountered when using transposons for identification is that they can move back and forth across the genome after complete cleavage, which poses difficulties in finding stable molecular markers. The number of reads at the transposon insertion site can intuitively reflect the necessity of a gene. Therefore, read counts are an important parameter in Tn seq analysis. When read counts equals 0, it indicates that the gene is an essential gene for bacterial growth. The larger the read counts value, the smaller the impact of the gene on bacterial growth. For example, the transposon insertion density of gene A is relatively high, and most insertion sites have high read counts, while under condition B, the results are opposite, indicating that this type of gene is called a conditionally necessary gene (McDonald,1993). Designing primers based on essential genetic differences can effectively identify different types of pathogens.

**Table 5:** Primer information for transposon detection technology of *F. oxysporum*.

Pathogen	Primer sequence (5′-3′)	Fragment size (bp)
*F. oxysporum*	Mg5:GGGGTCGGTTACATGGGTG	166
	Mg6:CAACAACAAGGCGAAGAGGG	
	Ha3F:CCCTCCAACATTCAACAACTG	187
	Ha3R:ATTCACTGTACACCAACCTTTT	

### 3.5 Pathogenicity-related genes based detection technology

Usually, the ability of fungi to infect specific vegetables depends on the unique genes encoding the host, which play important roles in the process of fungal infection. The differential factors that determine the toxicity of pathogenic bacteria include small mutations in a specific gene in the genome that controls the production of toxins by pathogenic bacteria. One of the first such tools developed for the forma special is *lycopersici*, based on a host-specific virulence gene. The gene encodes a small protein secreted in the xylem (*SIX*) that confers virulence to the fungus. Fourteen *SIX* genes are currently known, and a few homologs have been found in other forma speciales, such as cepae, cubense, and conglutinans (Li et al., 2016; Taylor et al., 2016) (Table 6). PCR primers were designed from *SIX* sequences to discriminate forma speciales cubense and lycopersici from other forma speciales (Fraser-Smith et al., 2014). Molecular markers based on other virulence factors were also designed for forma specialis phaseoli and for race 4 of forma specialis cubense (Aguayo et al., 2017). Lievens et al. (2009b) designed 7 pairs of tomato wilt pathogen-specific primers (*SIX1* ~ *SIX7*) based on nontoxic genes, and all of them could amplify specific fragments in *Fol* 1 ~ 3 (excluding *SIX4* primers). In FORL, none of the 7 pairs of primers were amplified. Zhang X. et al. (2023) and Zhang Y. et al. (2023) designed a specific primer FrlDel30 F/R based on the mutation site in the first chromosome of the nontoxic genes (*SIX1* ~ *SIX7*), which can effectively distinguish between root rot pathogens and it physiological races. Poueymiro et al. (2009) induced dual mutations in the non-toxic genes *avrA* and *popP1*, resulting different in pathogenic abilities of the bacterial strain (race 1), which is beneficial for distinguishing physiological races. Among them, *popP1* and *popP2* are located on the pathogenic island of 79 kb, while *popP3* is located on the pathogenic island of 83 kb (Xu et al., 2011). Among the analyzed proteomes of different Phytophthora species, Shands et al. (2024) found several orthogonal groups with the highest number of shared proteins (OG7457), and the conserved positive group only existed in the Pc2113 and Pc2109 isolates. Interestingly, a larger proportion of differentially expressed (DE) effectors were found in these orthogonal groups after pathogen infection, indicating that some of these effectors may play a conservative role in the pathogenicity of *Phytophthora*. The routine pathogen identification designs specific primers based on the differential loci between different genera of the pathogen, which is easy to operate and has obvious differential sequences, while the physiological race identification requires comparing the differential loci of virulence genes of the pathogen to design primers. Although knowledge on these genetic determinants was scarce until recently, it has considerably improved in the last decade.

**Table 6:** Primer information for pathogenicity-related genes technology of *F. oxysporum*.

Pathogen	Primer sequence (5′-3′)	Fragment size (bp)
*F. oxysporum*	SIX1-F:GTATCCCTCCGGATTTTGAGC	992
	SIX1-R:AATAGAGCCTGCAAAGCATG	
	SIX2-F:CAACGCCGTTTGAATAAGCA	749
	SIX2-R:TCTATCCGCTTTCTTCTCTC	
	SIX3-F:CCAGCCAGAAGGCCAGTTT	608
	SIX3-R:GGCAATAACCACTCTGCC	
	SIX4-F:TCAGGCTTCACTTAGCATAC	967
	SIX4-R:GCCGACCGAAAAACCCTAA	
	SIX5-F:ACACGCTCTACTACTCTTCA	667
	SIX5-R:GAAAACCTCAACGCGGCAAA	
	SIX6-F:CTCTCCTGAACCATCAACTT	793
	SIX6-R:CAAGACCAGGTGTAGGCATT	
	SIX7-F:CATCTTTTCGCCGACTTGGT	862
	SIX7-R:CTTAGCACCCTTGAGTAACT	
	FrlDel30F:GAGCGGGAGTTGAATTCTTG	204
	FrlDel30R:AAGAGCCTGCTCCA GTTGAA	

### 3.6 Genomics based detection technology

Recently, genomics technology has provided sequence information on dominant pathogenicity for the identification and analysis of physiological races of vegetable soil borne pathogens. A comparison of the entire genome suggests that the effect pool of each template may determine host specificity. However, comparative genomics is the next step to identify host specificity in *F oxysporum* (Van Dam et al., 2016). We performed whole genome sequencing of 45\u00B0*F. oxysporum* strains and managed to differentiate forma speciales *cucumerinum*, *niveum*, *melonis*, *radicis-cucumerinum*, and *lycopersici* on the basis of their effector pattern. Two years later, Van Dam et al. (2018) designed PCR primers to discriminate the seven forma speciales that affect *Cucurbitaceae* based on candidate effectors extracted from 82 genome assemblies. Zhang et al. (2014) used comparative genomics technique to design specific primers based on genome sequencing data of physiological races 1 and 2 of *Brassica oleracea*. They also confirmed the indeed differences in genome sequences between the two physiological races of *B. oleracea*. There is no doubt that expanding access to whole-genome sequences will continuously improve *F. oxysporum* host range identification. Therefore, for developing a rapid identification method for physiological races of pathogens is an ideal approach from the the perspective of searching for unique genes or fragments of different physiological races (Lievens et al., 2008). Meanwhile, the classification of physiological races of pathogens at the molecular level will be a major trend in future development.

## 4 Application of molecular detection techniques to detect pathogen load in soil

### 4.1 Pathogens load in soil

Soil is the main site for the growth and propagation of pathogenic or nonpathogenic microorganisms, resulting in continuous outbreaks of vegetable diseases (Yuan et al., 2020). Therefore, it is very important to identify and measure the levels of pathogens in soil. The occurrence and severity of diseases are positively related to the population of pathogens in soil; therefore, accurate measurement of the pathogen population in soil is the premise for disease forecasting and effective control. Huang C. H. et al. (2016) reported the design of a TaqMan probe and PCR primers for the DNA sequence of the species-specific virulence gene *SIX1*. Further results showed there was a significant positive correlation between the severity of soil borne disease and the concentration of *F. oxysporum* DNA in soil. Almquist et al. (2016) used RT–qPCR to quantitatively detect *Aphanomyces cochlioides* DNA in field soil samples and found that the bacterial content in clay was lower than that in sandy soil. Lastra et al. (2018) used RT–qPCR to determine that the DNA content of *F. solani* in diseased strawberry soil was between 16 and 190 pg.·mg^−1^, which became an effective tool for early warning and prevention of soil disease before plant transplantation. Zhong et al. (2022) developed an RT–qPCR assay for *F. oxysporum*, revealing that the total DNA of pathogens in soil after CaCN_2_ (240 and 300 mg·cm^3^) treatment decreased from 11.26 and 10.55 pg.·ng^−1^ to 4.21 and 4.01 pg./ng, respectively. Chen L. et al. (2022) determined that the *Fusarium* content of 8 out of 18 soil samples with *Fusarium* was 10^4^–10^6^ spores/g via PMA–qPCR, which provided a basis for the prediction of natural soil borne diseases. Chen L. D. et al. (2022) reported that fumigation with calcium cyanamide could lead to a relative reduction in the populations of soil pathogens, such as *Acremonium*, *Alternaria*, *Fusarium*, *Penicillium*, and *Verticillium,* using heterotrophic plate counts, PCR and MiSeq high-throughput sequencing. *C. cassiicola* is a potential pathogen in soil. The *C. cassiicola* content in soil after CaCN_2_ and plastic film treatment decreased from 10^7^ to 10^3^ spores·g^−1^ via PMA-qPCR detection method (Xie X. et al., 2022). Gu et al. (2022) revealed that tomato bacterial wilt was induced by isolation of the tomato rhizosphere microbial community. Disease diagnosis could be performed two weeks earlier based on the abundance of pathogenic bacteria causing tomato wilt in the rhizosphere microbial group using high-throughput sequencing technology.

### 4.2 Pathogens load in plants

Soil borne pathogen-infected vegetables showed root rot, browning, withering and root damage. The detection of pathogens in plants can be used in molecular biology research, such as for disease prediction, pathogen control and determination of pathogen interaction mechanisms. Gao et al. (2019) detected *V. dahliae* species and different metabolic substances in soil using macrogenomics technology, revealing the pathogenic mechanism underlying plant diseases. Meng et al. (2016) used RT–qPCR technology to detect *P. amygdali* pv. *lachrymans* in cucumber leaves, which allowed rapid and easy early assessment of angular leaf spot disease. Kuang et al. (2017) designed LAMP primers for *B. gladioli* pv. *alliicola* based on the *ITS* gene, which became an effective technique for detecting the pathogen in onion plants. Zhu et al. (2016) reported the development of an RT–qPCR assay based on the mitochondrial small subunit rDNA of *F. commune*, which will facilitate monitoring of the pathogen and improvised disease management. Kim et al. (2017) designed specific primers for *F. oxysporum* sp. *raphani* (FOR2-F/FOR2-R) and confirmed that the markers For_610_ and For_425_ could distinguish pathogenic *F. oxysporum* isolates. Klosterman et al. (2009) quantitatively detected the change in DNA content of Verticillium wilt *V. dahliae* by RNAseq technology and confirmed that *V. dahliae* infection was caused by wounds or cracks in the lateral roots of plants. Zhou et al. (2022) revealed that cross-kingdom (fungi and bacteria) synthetic communities (SynComs) were more effective in suppressing soil borne Fusarium wilt disease (FWD) than fungal or bacterial SynComs alone by plate isolation and culturing, RT–qPCR and high-throughput sequencing. Ten putative effectors were identified within FOC, including 7 *SIX* genes first reported in *F. oxysporum* f. sp. *lycopersici*, which can identify the types of pathogens in onion (Taylor et al., 2016). A specific combination of hydrolysis probes/primers has been developed using virulence genes, which can distinguish Foc race 4 (Aguayo et al., 2017). This new detection method can be used for plant regulatory detection applications.

### 4.3 Pathogens load in seeds

At present, there is a high demand for commercial vegetable seeds, leading to a higher probability of seeds carrying pathogens or long-distance transmission. Unlike the diagnosis of plant diseases, it is difficult to determine whether seeds carry pathogens because in most cases, infected seeds have no obvious symptoms of disease. In addition, the proportion of diseased seeds is small, and the distribution is uneven. All quarantine seeds do not conform to the actual situation. PCR technology is usually used for qualitative testing of most vegetable seed pathogens because it can detect even low DNA content of pathogens in diseased seeds and being discarded by the farm. Regarding, the occurrence of disease outbreaks and epidemics depends mainly on the number of seed carriers, suitability of the environment and host plant type (Lievens et al., 2008). Therefore, a sensitive, accurate and quantitative detection technology is needed to determine whether vegetable seeds carry pathogens to control the infection and spread of diseases from the root (Glynn and Edwards, 2010). The detection of vegetable seed-borne disease carriers requires a real-time detection technique with high sensitivity. The nested PCR-based technique could detect 32 conidia in 100 seeds within 4 h, which is suitable for commercial seed quarantine technology (Chiocchetti et al., 2001). Konstantinova et al. (2002) designed primers and confirmed that pathogens in carrot seeds contained pathogens such as *A. radicina*, *A. dauci*, and *A. alternata* using a PCR detection technique, which provided a favorable basis for the control of seed-borne vegetable diseases. Recently, some researchers have confirmed the existence of *A. brassicae* in cabbage and radish seeds by PCR and RT–qPCR techniques, where the pathogen DNA content was relatively high (Guillemette et al., 2004). The quantitative detection of *V. dahliae* in spinach seeds based on RT–qPCR allows the evaluation of seed infection rate up to 1.3%, providing a good technology for seed carrier quarantine and improved seed screening (Duressa et al., 2011). Sensitivity is very important in the detection of vegetable seed borne diseases. A new detection technique developed by Webb et al. (2014) has a sensitivity of 10 fg of pathogen DNA. Sousa et al. (2015) used RT–qPCR to detect and quantify the content of *F. oxysporum* f. sp. *phaseoli* in bean seeds, adding value to research on the spread of seed-borne pathogens. Tomato canker is a widespread and serious disease in vegetable production, especially due to the long-distance transmission of seed carriers that infect healthy plants. Wang et al. (2014) detected CMM in tomato seeds based on the RT–qPCR technique with high specificity and sensitivity. Ahmed et al. (2017) successfully isolated *Cladosporium* spp., *F. semitectum*, *F. oxysporum*, *Rhizoctonia* spp., and *Alternaria* from vegetable seeds by using standard blotter paper and the agar plate technique. This will be helpful for seed treatment with appropriate fungicides before sowing to overcome the loss caused by seed-borne fungi. A specific set of PCR primers was developed using IR-SCAR, which could uniquely amplify *F. oxysporum* race 1 from lettuce seeds in Italy, Portugal, the United States, Japan, and Taiwan (Sousa et al., 2015).

## 5 Conclusions and perspectives

Herein, we reviewed the recent progress of various molecular detection techniques for vegetable soil borne pathogens (PCR, nested-PCR, multiplex PCR, etc.) and their physiological races (host identification, DNA molecular markers, transposon detection, etc.), explaining the advantages and disadvantages of each detection technique. Furthermore, the paper comprehensively introduces the application of molecular detection technology in soil borne pathogen detection of soil, plants, and seeds. This paper will provide a value reference for future detection technique development for disease prevention and management of vegetable soil borne pathogen.

However, the applicability of soil borne pathogen and their physiological races detection is determined by technique sensitivity, planting variety, actual local conditions, etc. If possible, comparative genomics technology will be used in the further to analyze the entire genome data of various physiological races of soil borne pathogens, identify specific fragments and design specific primers to identify target strains, which will overcome the difficulty of identifying different physiological races of soil borne pathogens. Moreover, nowadays most of vegetables usually only have resistance to a certain physiological race of the pathogen, and this resistance may be overcome by more virulent physiological races, leading to more severe disease symptoms in the host crop. In the future more hosts will be used for pathogenicity assessment to screen vegetable insensitive or less-sensitive varieties to local dominant physiological race pathogens, thereby reducing economic losses and also provide important materials for disease resistance breeding (Pang et al., 2020; Schwelm and Ludwig-Muller, 2021). Furthermore, the objects polymorphism and adaptability of complex detection environments still need to be improved. It is necessary to focus on the ability of various detection methods to adapt to multi-objective and complex environments, improve the accuracy and efficiency. For researchers, the CRISPR/Cas12a method has its own advantages and good detection efficiency in detecting soil borne pathogens. For farmers, microscopic detection methods are more common and cost-effective. Therefore, precise detection of pathogens is not a single method. In practical applications, multiple methods need to be combined to improve the accuracy and reliability of detection. For example, preliminary detection can be conducted through a microscope, followed by validation and confirmation using the CRISPR/Cas12a method. The rapid progress of molecular detection technique for vegetables soil borne diseases will continuously promote the improvement of vegetable yield and quality, providing new vitality for green and sustainable development of the vegetable industry.

## References

[ref1] Abou-Jawdah Y. Aknadibossian V. Jawhari M. Tawidian P. Abrahamian P. (2019). Real-time PCR protocol for phytoplasma detection and quantifcation. Methods Mol. Biol. 1875, 117–130. doi: 10.1007/978-1-4939-8837-2_9, PMID: 30361999

[ref2] Achari S. R. Mann R. C. Sharma M. Edwards J. (2023). Diagnosis of *Fusarium oxysporum* f. sp. *ciceris* causing Fusarium wilt of chickpea using loop-mediated isothermal amplification (LAMP) and conventional end-point PCR. Sci. Rep. 13:2640. doi: 10.1038/s41598-023-29730-6, PMID: 36788315 PMC9929042

[ref3] Aguayo J. Mostert D. Fourrier-Jeandeal C. Cerf-Wendling I. Hostachy B. Viljoen A.. (2017). Development of hydrolysis probe-based real-time assay for the detection of tropical strains of *Fusarium oxysporum* f.sp.*cubense* race 4. PLoS One 12:e0171767. doi: 10.1371/journal.pone.0171767, PMID: 28178348 PMC5298334

[ref4] Ahmed B. Khan B. Ghazanfar M. U. Rajput N. A. Walait M. (2017). Occurrence and distribution of vegetables seed-borne mycoflora in Punjab Pakistan. Pak. J. Phytopathol. 29, 265–271. doi: 10.33866/phytopathol.029.02.0405

[ref5] Almasi M. A. (2019). Development of a colorimetric loop-mediated isothermal amplification assay for the visual detection of *fusarium oxysporum* f.sp. *melonis*. Horticult. Plant J. 5, 41–48. doi: 10.1016/j.hpj.2019.01.004

[ref6] Almquist C. Persson L. Olsson A. Sundstro M. J. Jonsson A. (2016). Disease risk assessment of sugar beet root rot using quantitative real-time PCR analysis of *aphanomyces cochlioides* in naturally infested soil samples. Eur. J. Plant Pathol. 145, 731–742. doi: 10.1007/s10658-016-0862-5

[ref7] Aruga D. Tsuchiya N. Matsumura H. Matsumoto E. Hayashida N. (2012). Analysis of RAPD and AFLP markers linked to resistance to *Fusarium oxysporum* f.sp. *lactucae* race 2 in lettuce (*Lactuca sativa* L.). Euphytica 187, 1–9. doi: 10.1007/s10681-012-0665-5

[ref8] Asano T. Senda M. Suga H. Kageyama K. (2010). Development of multiplex PCR to detect five *Pythium* species related to Turfgrass diseases. J. Phytopathol. 158, 609–615. doi: 10.1111/j.1439-0434.2009.01660.x

[ref9] Buczack S. T. Toxopeus H. Mattusch P. Johnston T. D. Hobolth L. A. (1975). Study of physiologic specialization in *Plasmodiophora brassicae*: proposals for attempted rationalization through an international approach. Trans. Br. Mycol. Soc. 65, 295–303. doi: 10.1016/S0007-1536(75)80013-1

[ref10] Cabanás C. G. L. Valverde Corredor A. Pérez Artés E. (2011). Molecular analysis of Spanish populations of *Fusarium oxysporum* f. sp. *dianthi* demonstrates a high genetic diversity and identifies virulence groups in races 1 and 2 of the pathogen. Eur. J. Plant Pathol. 132, 561–576. doi: 10.1007/s10658-011-9901-4

[ref11] Cao M. Q. Bao Q. Yang C. F. Wang J. Sheng S. Wu F. A. (2015a). Establishment of a qPCR method for rapid detect ion of *ralstonia solanacearum* race 5. Sci. Sericult. 41, 807–814. doi: 10.13441/j.cnki.cykx.2015.05.005

[ref12] Cao M. Q. Wang X. D. Wang J. Sheng S. Wu F. (2015b). Detection of living cells of *Ralstonia solanacearum* race 5 by PMA-qPCR method. Sci. Sericult. 41, 1004–1010. doi: 10.13441/j.cnki.cykx.2015.06.005

[ref13] Chai A. L. Ben H. Y. Guo W. T. Shi Y. X. Xie X. W. Li L.. (2020). Quantification of viable cells of *Pseudomonas syringae* pv. *Tomato* in tomato seed using propidium monoazide and a real-time PCR assay. Plant Dis. 104, 2225–2232. doi: 10.1094/PDIS-11-19-2397-RE 32452750

[ref14] Chang Y. D. Du B. Wang L. Ji P. Xie Y. J. Li X. F.. (2018). A study on the pathogen species and physiological races of tomato Fusarium wilt in Shanxi, China. J. Integr. Agricult. 17, 1380–1390. doi: 10.1016/S2095-3119(18)61983-5

[ref15] Chen Z. Halford N. G. Liu C. (2023). Real-time quantitative PCR: primer design, reference gene selection, calculations and statistics. Meta 13:806. doi: 10.3390/METABO13070806, PMID: 37512513 PMC10384377

[ref16] Chen L. Li L. Xie X. Chai A. Shi Y. Fan T.. (2022). An improved method for quantification of viable Fusarium cells in infected soil products by Propidium Monoazide coupled with real-time PCR. Microorganisms 10:1037. doi: 10.3390/MICROORGANISMS10051037, PMID: 35630479 PMC9143521

[ref17] Chen J. Ma E. Lucas B. Da C. Tian X. Palefsky J.. (2018). CRISPR-Cas12a target binding unleashes indiscriminate single-stranded DNase activity. Science 360, 436–439. doi: 10.1126/science.aar6245, PMID: 29449511 PMC6628903

[ref18] Chen L. D. Xie X. W. Kang H. J. Liu R. C. Shi Y. X. Li L.. (2022). Efficiency of calcium cyanamide on the control of tomato soil borne disease and their impacts on the soil microbial community. Appl. Soil Ecol. 176:104522. doi: 10.1016/J.APSOIL.2022.104522

[ref19] Chen L. D. Yuan J. H. Li L. Shi Y. X. Chai A. L. Xie X. W.. (2019). Development and application of quantitative PCR for detection of *Fusarium*. Acta Agricult. Boreali-Sinica 34, 296–301. doi: 10.7668/hbnxb.20190656 (in Chinese)

[ref20] Cheng Y. C. Kang H. J. Shi Y. X. Chai A. L. Zhang H. Xie X. W.. (2018). Development and application of real-time fluorescent quantitative PCR for detection of *Phytophthora capsici*. Acta Horticult. Sin. 45, 997–1006. doi: 10.16420/j.issn.0513-353x.2017-0862

[ref21] Chiocchetti A. Sciaudone I. Durando F. Garibaldi A. Migheli Q. (2001). PCR detection of *Fusarium oxysporum* f. sp. *basilici* on basil. Plant Dis. 85, 607–611. doi: 10.1094/PDIS.2001.85.6.607, PMID: 30823026

[ref22] Cho M. S. Lee J. H. Her N. H. Kim C. K. Seol Y. J. Hahn J. H.. (2012). A quantitative and direct PCR assay for the subspecies-specific detection of *Clavibacter michiganensis* subsp*. Michiganensis* based on a ferredoxin reductase gene. J. Microbiol. 50, 496–501. doi: 10.1007/s12275-012-1611-x, PMID: 22752914

[ref23] Cordova I. Oropeza C. Puch-Hau C. Harrison N. Colli-Rodríguez A. Narvaez M. A. (2014). Real-time PCR assay for detection of coconut lethal yellowing phytoplasmas of group 16SrIV subgroups a, D and E found in the Americas. J. Plant Pathol. 96, 343–352. doi: 10.4454/JPP.V96I2.031

[ref24] Crespo-Sempere A. Estiarte N. Marín S. Sanchis V. Ramos A. (2013). Propidium monoazide combined with real-time quantitative PCR to quantify viable *Alternaria* spp. contamination in tomato products. Int. J. Food Microbiol. 165, 214–220. doi: 10.1016/j.ijfoodmicro.2013.05.017, PMID: 23796654

[ref25] Ding X. Yin K. Li Z. Lalla R. V. Ballesteros E. Sfeir M. M.. (2020). Ultrasensitive and visual detection of SARS-CoV-2 using all-in-one dual CRISPR-Cas12a assay. Nat. Commun. 11:4711. doi: 10.1038/s41467-020-18575-6, PMID: 32948757 PMC7501862

[ref26] Duarte V. De Boer S. H. Ward L. J. de Oliveira A. M. R. (2004). Characterization of atypical *Erwinia carotovora* strains causing blackleg of potato in Brazil. J. Appl. Microbiol. 96, 535–545. doi: 10.1111/j.1365-2672.2004.02173.x, PMID: 14962133

[ref27] Duressa D. Rauscher G. Koike S. T. Mou B. Hayes R. J. Marutchachalam K.. (2011). A real-time PCR assay for detection and quantification of *Verticillium dahliae* in spinach seed. Phytopathology 102, 443–451. doi: 10.1094/PHYTO-10-11-0280, PMID: 22236050

[ref28] FAO (2023). Food and agricultural commodities production for 2023: Production of vegetable by countries [database.]. Rome: FAO Available at: http://faostat3.fao.org/browse/Q/QC/E.

[ref29] Foteini R. Jorge B. Alejandro G. Marta P. (2023). Real-time PCR, and recombinase polymerase amplification combined with SYBR green I for naked-eye detection, along with Propidium Monoazide (PMA) for the detection of viable patulin-producing fungi in apples and by-products. Food Control 144:109347. doi: 10.1016/J.FOODCONT.2022.109347

[ref30] Fraser-Smith S. Czislowski E. Meldrum R. A. Zander M. O'Neill W. Balali G. R.. (2014). Sequence variation in the putative effector gene *SIX*8 facilitates molecular differentiation of *Fusarium oxysporum* f.sp.*cubense*. Plant Pathol. 63, 1044–1052. doi: 10.1111/ppa.12184

[ref31] Fu H. T. Yang Y. L. Mishra V. Zhou Q. X. Zuzak K. Feindel D.. (2020). Most *Plasmodiophora brassicae* populations in single canola root galls from Alberta fields are mixtures of multiple strains. Plant Dis. 104, 116–120. doi: 10.1094/PDIS-06-19-1235-RE, PMID: 31644392

[ref32] Gao Y. Y. Wang N. Gao G. P. Wang W. (2010). Triplex PCR detection of *Cladosporium cucumerinum*, *Fusarium oxysporum* fsp. *Niveum* and *Mycosphaerella melonis* in infected plant tissues. Acta Phytopathol. Sin. 40, 343–350. doi: 10.13926/i.cnki.apps.2010.04016 (in Chinese)

[ref33] Gao S. G. Zeng R. Xu L. H. Luo J. Y. Chen L. Dai F. M. (2016). Triplex PCR detection for *Corynespora cassiicola*, *Colletotrichum orbiculare* and *Pseudomonas syringae* pv. *Lachrymans*. Sci. Agric. Sin. 49, 3119–3129. doi: 10.3864/j.issn.0578-1752.2016.16.006

[ref34] Gao F. Zhang B. S. Zhao J. H. Huang J. F. Jia P. S. Wang S.. (2019). Deacetylation of chitin oligomers increases virulence in soil borne fungal pathogens. Nat. Plants 5, 1167–1176. doi: 10.1038/s41477-019-0527-4, PMID: 31636399

[ref35] Geng L. H. Guo S. G. Lv G. Y. Zhang H. Y. Gong G. Y. Xu Y. (2010). Establishment and identification system for physiological races of *Fusarium oxyporum* f. sp. *niveum*. China Vegetables 1, 52–56. doi: 10.19928/j.cnki.1000-6346.2010.20.010 (in Chinese)

[ref36] Gharbi Y. Triki M. A. Trabelsi R. Fendri I. Daayf F. Gdoura R. (2015). Genetic structure of *Verticillium dahliae* isolates infecting olive trees in Tunisia using AFLP, pathogenicity and PCR markers. Plant Pathol. 64, 871–879. doi: 10.1111/ppa.12323

[ref37] Gieroń M. Żarnowiec P. Zegadło K. Gmiter D. Czerwonka G. Kaca W.. (2023). Loop-mediated isothermal amplification of DNA (LAMP) as an alternative method for determining Bacteria in wound infections. Int. J. Mol. Sci. 25:411. doi: 10.3390/IJMS25010411, PMID: 38203582 PMC10778741

[ref38] Glynn N. C. Edwards S. G. (2010). Evaluation of PCR assays for quantifying seed-borne infection by *Fusarium* and *Microdochium* seedling blight pathogens. J. Appl. Microbiol. 108, 81–87. doi: 10.1111/j.1365-2672.2009.04410.x, PMID: 20002908

[ref39] Grote D. Olmos A. Kofoet A. Tuset J. J. Bertolini E. Cambra M. (2002). Specific and sensitive detection of *Phytophthora nicotianae* by simple and nested-PCR. Eur. J. Plant Pathol. 108, 197–207. doi: 10.1023/A:1015139410793

[ref40] Gu Y. Samiran B. Francisco D. A. Xu Y. C. Shen Q. R. Alexandre J.. (2022). Small changes in rhizosphere microbiome composition predict disease outcomes earlier than pathogen density variations. ISME 16, 2448–2456. doi: 10.1038/s41396-022-01290-z, PMID: 35869387 PMC9478146

[ref41] Guillemette T. Iacomi-Vasilescu B. Simoneau P. (2004). Conventional and real-time PCR-based assay for detecting pathogenic *Alternaria brassicae* in *cruciferous* seed. Plant Dis. 88, 490–496. doi: 10.1094/PDIS.2004.88.5.490, PMID: 30812652

[ref42] Hirano Y. Arie T. (2006). PCR-based differentiation of *Fusarium oxysporum* f. sp. *lycopersici* and *radicis-lycopersici* and races of *F. Oxysporum f. sp. lycopersici*. J. Gen. Plant Pathol. 72, 273–283. doi: 10.1007/s10327-006-0287-7

[ref43] Hopkins A. J. M. Castanyo C. Boberg J. B. Stenlid J. (2013). Methods for the early detection of new invasive forest pathogens. Acta Phytopathol. Sin. 43:85.

[ref44] Htun M. Z. Rotchanapreeda T. Rujirawat T. Lohnoo T. Yingyong W. Kumsang Y.. (2020). Loop-mediated isothermal amplification (LAMP) for identification of *Pythium insidiosum*. Int. J. Infect. Dis. 101, 149–159. doi: 10.1016/j.ijid.2020.09.1430 32987181

[ref45] Huang C. H. Tsai R. T. E. Vallad G. (2016). Development of a TaqMan real-time polymerase chain reaction assay for detection and quantification of *Fusarium oxysporum* f. sp. *lycopersici* in soil. J. Phytopathol. 164, 455–463. doi: 10.1111/jph.12471

[ref46] Huang W. Xu J. Zhang H. Xu J. S. Ding W. Feng J. (2016). Development of a LAMP approach for detection of *Ralstonia solanacearum*. Sci. Agric. Sin. 49, 2093–2102. doi: 10.3864/j.issn.0578-1752.2016.11.006

[ref47] Israa A. H. Hayder H. I. Aleem M. K. (2023). A review of the development in the multiplex PCR technique for the detection of *Bacillus cereus*. ESS Open Arch. 29:2023. doi: 10.22541/au.168006062.20963062/v1

[ref48] Jambhulkar P. P. Sharma M. Lakshman D. Sharma P. (2015). “Natural mechanisms of soil suppressiveness against diseases caused by Fusarium, Rhizoctonia, Pythium, and Phytophthora” in Organic amendments and soil suppressiveness in plant disease management. eds. Meghvansi M. K. Varma A. (New York: Springer), 95–124.

[ref49] Jesús M. B. Dolores R. J. Encarnación P.-A. Rafael M. J. D. (2002). Detection of the defoliating Pathotype of *Verticillium dahliae* in infected olive plants by nested PCR. Eur. J. Plant Pathol. 50, 609–619. doi: 10.1046/j.1365-3059.2001.00601.x

[ref50] Ji P. S. Allen C. Sanchez-Perez A. Yao J. Elphinstone J. G. Jones J. B. (2007). New diversity of *Ralstonia solanacearum* strains associated with vegetable and ornamental crops in Florida. Plant Dis. 91, 195–203. doi: 10.1094/PDIS-91-2-0195, PMID: 30781004

[ref51] Jones D. R. Ingram D. S. Dixon G. R. (1982). Factors affecting tests for differential pathogenicity in populations of *Plasmodiophora brassicae*. Plant Pathol. 31, 229–238. doi: 10.1111/j.1365-3059.1982.tb01273.x

[ref52] Kang H. J. Chai A. L. Shi Y. X. Xie X. W. Yuan J. H. Li B. J. (2018). Quadruple PCR detection of *Pseudomonas syringae* pv. Tomato, *Clavibacter michiganensis* subsp. *michiganensis*, *Ralstonia solanacearum* and *Xanthomonas campestris* pv. *Vesicatoria* in infected tomato tissues. Acta Horticult. Sin. 45, 2255–2264. doi: 10.16420/j.issn.0513-353x.2018-0153

[ref53] Kang H. X. Peng Y. Hua K. Y. Deng Y. F. Bellizzi M. Gupta D. R.. (2021). Rapid detection of wheat blast pathogen *Magnaporthe oryzae triticum* pathotype using genome-specific primers and Cas12a-mediated technology. Engineering 7, 1326–1335. doi: 10.1016/j.eng.2020.07.016

[ref54] Khan M. Li B. Jiang Y. Weng Q. Chen Q. (2017). Evaluation of different PCR-based assays and LAMP method for rapid detection of *Phytophthora infestans* by targeting the Ypt1 gene. Front. Microbiol. 8:1920. doi: 10.3389/fmicb.2017.01920, PMID: 29051751 PMC5633602

[ref55] Khat M. N. Nisachon J. Lapatrada T. (2024). Evaluation of molecular inhibitors of loop-mediated isothermal amplification (LAMP). Sci. Rep. 14:5916. doi: 10.1038/S41598-024-55241-Z, PMID: 38467647 PMC10928092

[ref56] Kim H. Hwang S. M. Lee J. H. Oh M. Han J. W. Choi G. J. (2017). Specific PCR detection of *Fusarium oxysporum* f. sp. *raphani*: a causal agent of Fusarium wilt on radish plants. Lett. Appl. Microbiol. 65, 133–140. doi: 10.1111/lam.12761, PMID: 28585248

[ref57] Kim D. Jeong R. D. Choi S. Ju H. J. Yoon J. Y. (2023). Application of rapid and reliable detection of *Cymbidium mosaic virus* by reverse transcription recombinase polymerase amplification combined with lateral flow immunoassay. Plant Pathol. J. 39:158. doi: 10.5423/PPJ.ER.10.2022.0147, PMID: 36760058 PMC9929169

[ref58] Klemsdal S. S. Elen O. (2006). Development of a highly sensitive nested-PCR method using a single closed tube for detection of *Fusarium culmorum* in cereal samples. Lett. Appl. Microbiol. 42, 544–548. doi: 10.1111/j.1472-765X.2006.01880.x, PMID: 16620217

[ref59] Klosterman S. J. Atallah Z. K. Vallad G. E. Subbarao K. V. (2009). Diversity, pathogenicity, and management of *verticillium* species. Annu. Rev. Phytopathol. 47, 39–62. doi: 10.1146/annurev-phyto-080508-081748 19385730

[ref60] Koentjoro M. P. Hidayat T. Maat S. Fasya A. H. Prasetyo E. N. (2023). Development of nested-PCR assay for the rapid detection of *Aspergillus fumigatus*. In AIP conference. 2634, AIP publishing.

[ref61] Konstantinova P. Bonants P. J. M. Gent-Pelzer M. P. E. V. Zouwen P. V. D. Bulk R. V. D. (2002). Development of specific primers for detection and identification of *Alternaria* spp. in carrot material by PCR and comparison with blotter and plating assays. Mycol. Res. 106, 23–33. doi: 10.1017/S0953756201005160

[ref62] Kuang R. R. Lei R. Jiang L. Duan W. J. Li X. L. Fu N.. (2022). Establishment of RPA/CRISPR-Cas12a rapid detection for *Leptosphaeria lindquistii*. Plant Prot. 48, 69–76. doi: 10.16688/j.zwbh.2022401 (In Chinese)

[ref63] Kuang W. L. Luo L., Gao W. N., Lei Y. H. Lv Q. Y. Li J. Q. (2017). Development of a real-time fluorescence loop-mediated isothermal amplification assay for detection of *Burkholderia gladioli* pv. *Alliicola*. J. Phytopathol. 165, 82–90. doi: 10.1111/jph.12539

[ref64] Lan C. Z. Liu P. Q. Li B. J. Chen Q. H. Weng Q. Y. (2013). Development of a specific pcr assay for the rapid and sensitive detection of *Phytophthora capsici* Australas. Plant Pathol. 42, 379–384. doi: 10.1007/s13313-012-0185-8

[ref65] Lastra E. Basallote-Ureba M. J. Santos B. Miranda L. Vela-Delgado M. D. Capote N. (2018). A TaqMan real-time polymerase chain reaction assay for accurate detection and quantification of *Fusarium solani* in strawberry plants and soil. Sci. Hortic. 237, 128–134. doi: 10.1016/j.scienta.2018.04.007

[ref66] Lecomte C. Edel-Hermann V. Cannesan M. A. Gautheron N. Langlois A. Alabouvette C.. (2016). *Fusarium oxysporum* f.sp.*cyclaminis*: underestimated genetic diversity. Eur. J. Plant Pathol. 145, 421–431. doi: 10.1007/s10658-016-0856-3

[ref67] Lei R. Sun X. W. Jiang L. Wang Z. H. Li G. Q. Li Y.. (2022). Development of rapid detection for *Phytophthora syringae* based on RPA/CRISPR-Cas12a. Plant Quarantine 36, 31–38. doi: 10.19662/j.cnki.issn1005-2755.2022.03.006 (In Chinese)

[ref68] Li B. J. Liu P. Q. Xie S. Y. Yin R. M. Weng Q. Y. Chen Q. H. (2014). Specific and sensitive detection of *phytophthora nicotianae* by nested pcr and loop-mediated isothermal amplification assays. J. Phytopathol. 163, 185–193. doi: 10.1111/jph.12305

[ref69] Li E. Wang G. Xiao J. Ling J. Yang Y. Xie B. (2016). A SIXI homolog in *Fusarium oxysporum* f.sp. *conglutinans* is required for full virulence on cabbage. PLoS One 11:e0152273. doi: 10.1371/journal.pone.0152273, PMID: 27010418 PMC4807099

[ref70] Li X. J. Zhang S. Y. Liu D. Yuan X. W. Li X. S. Shi Y. X.. (2022). Establishment and application of rapid quantitative detection of viable *Plasmodiophora brassicae* by PMAxx-qPCR method. Sci. Agric. Sin. 55, 1938–1948. doi: 10.3864/j.issn.0578-1752.2022.10.005

[ref71] Li J. Zhao W. Wang J. X. Zhou M. G. (2011). Detection of *Sclerotinia sclerotiorum* by a quantitativereal-time PCR. Acta Phytopathol. Sin. 185, 5828–5834. doi: 10.4049/jimmunol.0903636, PMID: 20956337 PMC4355963

[ref72] Lievens B. Houterman P. Rep M. (2009a). Effector gene screening allows unambiguous identification of *Fusarium oxysporum* f.sp *lycopersici* races and discrimination from other formae speciales. FEMS Microbiol. Lett. 300, 201–215. doi: 10.1111/j.1574-6968.2009.01783.x, PMID: 19799634

[ref73] Lievens B. Rep M. Thomma B. P. (2008). Recent developments in the molecular discrimination of formae speciales of *Fusarium oxysporum*. Pest Manag. Sci. 64, 781–788. doi: 10.1002/ps.1564, PMID: 18335459

[ref74] Lievens B. van Baarlen P. Verreth C. van Kerckhove S. Rep M. Thomma B. P. H. J. (2009b). Evolutionary relationships between *Fusarium oxysporum* f. sp. *lycopersici* and *F. Oxysporum* f. sp. *radicis-lycopersici* isolates inferred from mating type, elongation factor-1α and exopolygalacturonase sequences. Mycol. Res. 113, 1181–1191. doi: 10.1016/j.mycres.2009.07.019 19679185

[ref75] Lin Y. H. Chang J. Y. Liu E. T. Chao C. P. Huang J. W. Chang P. F. L. (2008). Development of a molecular marker for specific detection of *Fusarium oxysporum* f.sp. *cubense* race 4. Eur. J. Plant Pathol. 123, 353–365. doi: 10.1007/s10658-008-9372-4

[ref76] Liop P. Bonaterra A. Peñalver J. López M. M. (2000). Development of a highly sensitive nested-PCR procedure using a single closed tube for detection of *Erwinia amylovora* in asymptomatic plant material. Appl. Environ. Microbiol. 66, 2071–2078. doi: 10.1128/AEM.66.5.2071-2078.2000, PMID: 10788384 PMC101457

[ref77] Liu R. C. Cheng Y. P. Chai A. L. Shi Y. X. Xie X. W. Patiguli L. B. J. (2019). Establishment and application of a triplex PCR detection system for vegetable soil borne pathogens. Sci. Agric. Sin. 52, 2069–2078. doi: 10.3864/j.issn.0578-1752.2019.12.005

[ref78] Liu Y. Ji Y. Han Y. C. Song L. L. Duan K. (2021). Loop-mediated isothermal amplification and PCR combined assay to detect and distinguish latent *Colletotrichum* spp. infection on strawberry. J. Plant Pathol. 103, 1–13. doi: 10.1007/S42161-021-00873-7

[ref79] Liu H. Wang J. B. Zeng H. J. Liu X. F. Jiang W. Wang Y.. (2021). RPA-Cas12a-FS: a frontline nucleic acid rapid detection system for food safety based on CRISPR-Cas12a combined with recombinase polymerase amplification. Food Chem. 334:127608. doi: 10.1016/j.foodchem.2020.127608, PMID: 32711280

[ref80] López-Berges M. S. di Pietro A. Daboussi M. J. Wahab H. A. Vasnier C. Roncero M. I.. (2009). Identification of virulence genes in *Fusarium oxysporum* f.sp. *lycopersici* by large-scale transposon tagging. *Mol*. Plant Pathol. 10, 95–107. doi: 10.1111/j.1364-3703.2008.00512.x, PMID: 19161356 PMC6640436

[ref81] Lu C. Song B. Zhang H. F. Wang Y. C. Zheng X. B. (2015). Rapid diagnosis of soybean seedling blight caused by *rhizoctonia solani* and soybean charcoal rot caused by *macrophomina phaseolina* using lamp assays. Phytopathology 105, 1612–1617. doi: 10.1094/PHYTO-01-15-0023-R, PMID: 26606587

[ref82] Luo L. X. Walters C. Bolkan H. Liu X. L. Li J. Q. (2008). Quantification of viable cells of *Clavibacter michiganensis* subsp. *michiganensis* using a DNA binding dye and a real-time PCR assay. Plant Pathol. 57, 332–337. doi: 10.1111/j.1365-3059.2007.01736.x

[ref83] Manzanares-dauleux M. J. Barret P. Thomas G. (2000). Development of a pathotype specific SCAR marker in *Plasmodiophora brassicae*. Eur. J. Plant Pathol. 106, 781–787. doi: 10.1023/A:1026586803761

[ref84] Martinez-Culebras P. V. Querol A. Suarez-Fernandez M. B. Garcia-Lopez M. D. Barrio E. (2003). Phylogenetic relationships among *Colletotrichum* pathogens of strawberry and design of PCR primer for their identification. Phytopathology 151, 135–143. doi: 10.1046/j.1439-0434.2003.00694.x

[ref85] Martyn R. D. Netzer D. (1991). Resistance to races 0, 1, and 2 of Fusarium wilt of watermelon in *Citrullus* sp. PI296341-FR. HortScience 26, 429–432. doi: 10.21273/HORTSCI.26.4.429

[ref86] Matsumoto M. (2002). Trials of direct detection and identification of *Rhizoctonia solani* AG1 and AG2 subgroups using specifically primed PCR analysis. Mycoscience 43, 185–189. doi: 10.1007/s102670200026

[ref87] Mbofung G. C. Y. Pryor B. M. (2010). A PCR-based assay for detection of *Fusarium oxysporum* f. sp. *lactucae* in lettuce seed. Plant Dis. 94, 860–866. doi: 10.1094/PDIS-94-7-0860, PMID: 30743548

[ref88] McDonald J. F. (1993). Evolution and consequences of transposable elements. Curr. Opin. Genet. Dev. 3, 855–864. doi: 10.1016/0959-437X(93)90005-A 8118210

[ref89] Meng X. L. Chai A. L. Chen L. Shi Y. X. Xie X. W. Ma Z. H.. (2016). Rapid detection and quantification of viable *Pseudomonassyringae* pv. *Lachrymans* cells in contaminated cucumber seeds using propidium monoazide and a real-time PCR assay. Can. J. Plant Pathol. 38, 296–306. doi: 10.1080/07060661.2016.1216897

[ref90] Mirmajlessi S. M. Loit E. Maend M. Mansouripour S. M. (2015). Real time PCR applied to study on plant pathogens: potential applications in diagnosis-a review. Plant Prot. Sci. 51, 177–190. doi: 10.17221/104/2014-PPS

[ref91] Mudiyanselage A. M. H. Jones E. E. Jaspers M. V. Walter M. Ridgway H. J. (2021). A one step nested pcr method for detection of *peronospora sparsa*, the downy mildew pathogen, in boysenberry (*rubus ursinus*). Eur. J. Plant Pathol. 160, 973–981. doi: 10.1007/s10658-021-02283-y

[ref93] Murolo S. Moumni M. Mancini V. Allagui M. B. Landi L. Romanazzi G. (2022). Detection and quantification of *Stagonosporopsis cucurbitacearum* in seeds of *Cucurbita maxima* using droplet digital polymerase chain reaction. Front. Microbiol. 12:764447. doi: 10.3389/FMICB.2021.764447, PMID: 35087483 PMC8788924

[ref94] Mutasa E. S. Chwarszcynska D. M. Asher M. J. C. (1996). Single-tube, nested PCR for the diagnosis of Polymyxa betae infection in sugar beet roots and colorimetric analysis of amplified products. Phytopathology 86, 493–497. doi: 10.1094/Phyto-86-493

[ref95] Nair S. Manimekalai R. (2021). Phytoplasma diseases of plants: molecular diagnostics and way forward. World J. Microbiol. Biotechnol. 37:102. doi: 10.1007/S11274-021-03061-Y, PMID: 34009500

[ref96] Nocker A. Cheung C. Y. Camper A. K. (2006). Comparison of propidium monoazide with ethidium monoazide for differentiation of live vs. dead bacteria by selective removal of DNA from dead cells. J. Microbiol. Methods 67, 310–320. doi: 10.1016/j.mimet.2006.04.015, PMID: 16753236

[ref97] O'Donnell K. Sutton D. A. Rinaldi M. G. Sarver B. A. J. Geiser D. M. (2010). Internet-accessible DNA sequence database for identifying Fusaria from human and animal infections. J. Clin. Microbiol. 48, 3708–3718. doi: 10.1128/jcm.00989-10, PMID: 20686083 PMC2953079

[ref98] O'Donnell K. Ward T. J. Robert V. A. R. G. Crous P. W. Geiser D. M. Kang S. (2015). DNA sequence-based identification of *Fusarium*: current status and future directions. Phytoparasitica 43, 583–595. doi: 10.1007/s12600-015-0484-z

[ref99] Oliveira M. B. Nascimento L. B. Junior M. L. Petrofeza S. (2010). Characterization of the dry bean polygalacturonase-inhibiting protein (pgip) gene family during *Sclerotinia sclerotiorum* (*sclerotiniaceae*) infection. GMR 9, 994–1004. doi: 10.4238/vol9-2gmr776, PMID: 20533194

[ref100] Olmos A. Bertolini E. Martınez M. C. Candresse T. Glasa M. Pina J. A.. (2013). Deep sequencing: a powerful tool for detection and characterization of known and new plant viruses and viroids. Acta Phytopathol. Sin. 43:280.

[ref101] Ortu G. Bertetti D. Gullino M. L. Garibaldi A. (2018). *Fusarium oxysporum* f. sp. *lavandulae*, a novel forma specialis causing wilt on *Lavandula* × *allardii*. J. Plant Pathol. 100, 391–397. doi: 10.1007/s42161-018-0084-0

[ref102] Ozdemir Z. (2005). Development of a multiplex PCR assay for concurrent detection of *Clavibacter michiganensis* ssp. *michiganensis* and *Xanthomonas axonopodis* pv. *Vesicatoria*. Plant Pathol. J. 4, 133–137. doi: 10.3923/ppj.2005.133.137

[ref103] Pace N. R. (1997). A molecular view of microbial diversity and the biosphere. Science 276, 734–740. doi: 10.1126/science.276.5313.734, PMID: 9115194

[ref104] Pang W. X. Liang Y. Zhan Z. X. Li X. N. Piao Z. Y. (2020). Development of a Sinitic clubroot differential set for the pathotype classification of *Plasmodiophora brassicae*. Front. Plant Sci. 11:568771. doi: 10.3389/fpls.2020.568771, PMID: 32983217 PMC7488845

[ref105] Panno S. Ferriol I. Rangel E. Olmos A. Han C. G. Martinelli F.. (2014). Detection and identification of *Fabavirus* species by one-step RT-PCR and multiplex RT-PCR. J. Virol. Methods 197, 77–82. doi: 10.1016/j.jviromet.2013.12.002, PMID: 24361876

[ref106] Pasquali M. Dematheis F. Gullino M. L. Garibaldi A. (2007). Identification of race 1 of *Fusarium oxysporum* f. sp. *lactucae* on lettuce by inter-retrotransposon sequence-characterized amplified region technique. Phytopathology 97, 987–996. doi: 10.1094/PHYTO-97-8-0987, PMID: 18943639

[ref107] Pasquali M. Marena L. Fiora E. Piatti P. Gullino M. L. Garibaldi A. (2004). Real time PCR for the identification of a highly pathogenic group of *Fusarium oxysporum* f. sp. *chrysanthemi* on *Argyranthemum frutescens* L. J. Plant Pathol. 86, 51–57. doi: 10.2307/41998167

[ref108] Pastrik K. H. Elphinstone J. G. Pukall R. (2002). Sequence analysis and detection of *Ralstonia solanacearum* by multiplex PCR amplification of 16S-23S ribosomal intergenic spacer region with internal positive control. Eur. J. Plant Pathol. 108, 831–842. doi: 10.1023/A:1021218201771

[ref109] Peng J. Zhan Y. F. Zeng F. Y. Long H. B. Pei Y. L. Guo J. R. (2013). Development of a real-time fluorescence loop-mediated isothermal amplification assay for rapid and quantitative detection of *Fusarium oxysporum* f. sp*. niveum* in soil. FEMS Microbiol. Lett. 349, 127–134. doi: 10.1111/1574-6968.12305, PMID: 24256412

[ref110] Poueymiro M. Cunnac S. Barberis P. Deslandes L. Peeters N. Cazale-Noel A. C.. (2009). Two type III secretion system effectors from *Ralstonia solanacearum* GMI1000 determine host range specificity on tobacco. Mol. Plant Microbe Interact. 22, 538–550. doi: 10.1094/MPMI-22-5-0538, PMID: 19348572

[ref111] Qin L. Fu Y. Xie J. Cheng J. Jiang D. Li G.. (2011). A nested-PCR method for rapid detection of *Sclerotinia sclerotiorum* on petals of oilseed rape (*Brassica napus*). Plant Pathol. 60, 271–277. doi: 10.1111/j.1365-3059.2010.02372.x

[ref112] Quinterovásquez G. A. Bazántejeda M. L. Martínezpeñafiel E. Kameyamakawabe L. Bermúdez-Cruz R. M. (2010). Multiplex PCR to detect four different tomato-infecting pathogens. Folia Microbiol. 58, 269–276. doi: 10.1007/s12223-012-0206-6, PMID: 23135900

[ref113] Ramesh R. Anthony J. Jaxon T. C. D. Gaitonde S. Achari G. (2011). PCR-based sensitive detection of *Ralstonia solanacearum* from soil, eggplant, seeds and weeds. Arch. Phytopathol. Plant Protect. 44, 1908–1919. doi: 10.1080/03235408.2010.516087

[ref114] Rui T. T. Gao Q. Y. Li X. J. Shi Y. X. Xie X. W. Li L.. (2022). Methylene blue combined agarose assay for single-spore isolation of *Plasmodiophora brassicae* and pathotype differentiation. Acta Horticult. Sin. 49, 1290–1300. doi: 10.16420/j.issn.0513-353x.2021-0421

[ref115] Schwelm A. Ludwig-Muller J. (2021). Molecular pathotyping of *Plasmodiophora brassicae*-genomes, marker genes, and obstacles. Pathogens 10:259. doi: 10.3390/pathogens10030259, PMID: 33668372 PMC7996130

[ref116] Shands A. C. Xu G. Belisle R. J. Seifbarghi S. Jackson N. Bombarely A.. (2024). Genomic and transcriptomic analyses of *Phytophthora cinnamomi* reveal complex genome architecture, expansion of pathogenicity factors, and host-dependent gene expression profiles. Front. Microbiol. 15:1341803. doi: 10.3389/fmicb.2024.1341803, PMID: 39211322 PMC11357935

[ref117] Shneyder Y. Karimova E. Zhivaeva T. Lozovaya E. Bashkirova I. Prikhodko Y. (2022). Comparison of express-diagnostic kit and PCR tests for detection of *tomato brown rugose fruit virus*. Acta Hortic. 1351, 113–118. doi: 10.17660/ActaHortic.2022.1351.18

[ref118] Sousa M. Machado J. Simmons H. E. Munkvold G. P. (2015). Real-time quantitative pcr assays for the rapid detection and quantification of *fusarium oxysporum* f. sp. *Phaseoli* in *phaseolus vulgaris* (common bean) seeds. Plant Pathol. 64, 478–488. doi: 10.1111/ppa.12257

[ref119] Supakitthanakorn S. Vichittragoontavorn K. Sunpapao A. Kunasakdakul K. Thapanapongworakul P. Ruangwong O. U. (2022). Tobacco mosaic virus infection of chrysanthemums in Thailand: development of colorimetric reverse-transcription loop-mediated isothermal amplification (RT–LAMP) technique for sensitive and rapid detection. Plan. Theory 11:1788. doi: 10.3390/PLANTS11141788, PMID: 35890422 PMC9325109

[ref120] Tavernier S. Coenye T. (2015). Quantification of *Pseudomonas aeruginosa* in multispecies biofilms using PMA-qPCR. PeerJ 3:e787. doi: 10.7717/peerj.787, PMID: 25755923 PMC4349053

[ref121] Taylor A. Vagany V. Jackson A. C. Harrison R. J. Rainoni A. Clarkson J. P. (2016). Identification of pathogenicity-related genes in *Fusarium oxysporum* f.sp.*cepae*. Mol. Plant Pathol. 17, 1032–1047. doi: 10.1111/mpp.12346, PMID: 26609905 PMC4982077

[ref122] Tian Q. Feng J. J. Hu J. Zhao W. J. (2016). Selective detection of viable seed-borne *Acidovorax citrulli* by real-time PCR with propidium monoazide. Sci. Rep. UK 6:35457. doi: 10.1038/srep35457, PMID: 27739469 PMC5064318

[ref123] Umesha S. Avinash P. (2015). Multiplex PCR for simultaneous identification of *Ralstonia solanacearum* and *Xanthomonas perforans*. Biotech 5, 245–252. doi: 10.1007/s13205-014-0223-z, PMID: 28324289 PMC4434412

[ref124] Van Dam P. de Sain M. ter Horst A. van der Gragt M. Rep M. (2018). Use of comparative genomics-based markers for discrimination of host specificity in *Fusarium oxysporum*. Appl. Environ. Microbiol. 84, e01868–e01817. doi: 10.1128/AEM.01868-17, PMID: 29030446 PMC5734036

[ref125] Van Dam P. Fokkens L. Schmidt S. M. Linmans J. H. J. Kistler H. C. Ma L. J.. (2016). Effect or profiles distinguish formae speciales of *Fusarium oxysporum*. Environ. Microbiol. 18, 4087–4102. doi: 10.1111/1462-2920.13445 27387256

[ref126] Vojvodic M. Lazic D. Mitrovic P. Tanovic B. Bulajic A. (2019). Conventional and real-time PCR assays for detection and identification of *Rhizoctonia solani* AG-2-2, the causal agent of root rot of sugar beet. Pesticidi i Fitomedicina 34, 19–29. doi: 10.2298/PIF1901019V

[ref127] Wang N. W. Wang T. Shen J. G. Hu F. P. (2014). Rapid detection for *Clavibacter michiganensis* subsp. *michiganensis* using real-time PCR based on padlock probe. Sci. Agric. Sin. 47, 903–911. doi: 10.3864/j.issn.0578-1752.2014.05.007

[ref128] Wang B. Wang R. Wang D. Q. Wu J. Li J. X. Wang J.. (2019). Cas12aVDet: a CRISPR/Cas12a-based platform for rapid and visual nucleic acid detection. Anal. Chem. 91, 12156–12161. doi: 10.1021/acs.analchem.9b01526, PMID: 31460749

[ref129] Wang X. Zhong M. Liu Y. Ma P. X. Liu M. (2020). Rapid and sensitive detection of COVID-19 using CRISPR/Cas12a- based detection with naked eye readout, CRISPR/Cas12a-NER. Sci. Bull. 65, 1436–1439. doi: 10.1016/j.scib.2020.04.041, PMID: 32373393 PMC7198415

[ref130] Webb K. Gilardi G. Matic S. Gullino M. L. Garibaldi A. (2014). Development of a rapid in planta detection method for identifying *Plectosphaerella cucumerina* on wild rocket by real-time PCR. Eur. J. Plant Pathol. 47, 903–911. doi: 10.14601/PhytopatholMediterr-15108

[ref131] Wei Y. K. Wang Q. Xu X. M. Hu X. P. Shang W. J. (2024). Development and application of rapid detection system for *Fusarium pseudograminearum* based on RPA-CRISPR/Cas12a. Mycosystema 43:240061. doi: 10.13346/j.mycosystema.240061

[ref132] Willer H. Meier C. Schlatter B. Travnicek J. (2023). The state and evolution of organic fruit and vegetables: production and market at a world scale. Acta Hortic. 1367, 1–12. doi: 10.17660/ActaHortic.2023.1367.1

[ref133] Williams P. H. (1966). A system for the determination of races of *Plasmodiophorae brassicae* that infect cabbage and rutabaga. Phytopathology 56, 624–626.

[ref134] Wu X. H. Shao X. L. Deng M. J. Chen C. F. Li Y. Liang C. Z. (2007). Study on rapid detection of *Clavibacter michiganensis* subsp.*michiganensis* by real-time fluorescent PCR. Acta Agricult. Jiangxi 19, 34–36. doi: 10.19386/j.cnki.jxnyxb.2007.03.012 (in Chinese)

[ref135] Xie X. Chen L. Shi Y. Chai A. Fan T. Li L.. (2022). The calcium cyanamide and polyethylene blocks the secondary transmission and infection of vegetable leaf diseases. Front. Plant Sci. 13:1027584. doi: 10.3389/FPLS.2022.1027584, PMID: 36605967 PMC9807914

[ref136] Xie X. Chen L. Shi Y. Chai A. Fan T. Li B.. (2024). Response of microbial recovery rate to straw return after calcium Cyanamide soil disinfection. Horticulturae 10:2. doi: 10.3390/horticulturae10010002

[ref137] Xie X. W. Liu S. C. Shi Y. X. Zhang S. F. Chen L. D. Chai A. L.. (2022). Establishment and application of LAMP rapid detection assay for *stephylium solani*. J. Agricult. Biotechnol. 30, 2045–2052. doi: 10.3969/j.issn.1674-7968.2022.10.018

[ref138] Xu J. Zheng H. J. Liu L. Pan Z. C. Prior P. Tang B.. (2011). Complete genome sequence of the plant pathogen *Ralstonia solanacearum* strain Po82. J. Bacteriol. 193, 4261–4262. doi: 10.1128/JB.05384-11, PMID: 21685279 PMC3147697

[ref139] Yang J. Duan K. Liu Y. Song L. Gao Q. (2022). Method to detect and quantify colonization of anthracnose causal agent *Colletotrichum gloeosporioides* species complex in strawberry by real-time PCR. J. Phytopathol. 170, 326–336. doi: 10.1111/jph.13082

[ref140] Yao X. Li P. Xu J. Zhang M. Ren R. Liu G.. (2016). Rapid and sensitive detection of Didymella bryoniae by visual loop-mediated isothermal amplification assay. Front. Microbiol. 7:1372. doi: 10.3389/fmicb.2016.01372, PMID: 27625648 PMC5003822

[ref141] Ye Q. J. Wang R. Q. Ruan M. Y. Cheng Y. Wan H. J. Li Z. M.. (2022). Establishment of a KASP-SNP detection method for filamentous fungi *Fusarium oxysporum* f. sp. *lycopersici* and *Fusarium oxysporum* f. sp. *radicis lycopersici*. J. Plant Prot. 49, 879–889. doi: 10.13802/j.cnki.zwbhxb.2022.2020180

[ref142] Yi C. L. Zheng J. Fu Y. D. Sun Q. Y. Jing C. Fang X. Y.. (2020). The specific molecular marker for identification of *Plasmodiophora brassicae* pathotype 4. J. Sichuan Agricult. Univ. 38:6. doi: 10.16036/j.issn.1000-2650.2020.03.008 (in Chinese)

[ref143] Youssef S. A. Sayed Y. Hassan O. S. Safwat G. Shalaby A. A. (2017). Universal and specifc 16S-23Sr RNA PCR primers for identification of phytoplasma associated with sesame in Egypt. Int. J. Adv. Res. Biol. Sci. 4, 191–200. doi: 10.22192/ijarbs.2017.04.07.024

[ref144] Yu X. Wang W. F. Li X. F. Feng L. X. Qian Y. K. Zhang C. J.. (2021). PMA-qPCR assay for the detection of viable *Pseudomonas syringae* pv. *Maculicola*. Plant Quarantine 35, 49–54. doi: 10.19662/j.cnki.issn1005-2755.2021.04.009

[ref145] Yuan J. Wen T. Zhang H. Zhao M. L. Penton C. Ryan S.. (2020). Predicting disease occurrence with high accuracy based on soil macroecological patterns of Fusarium wilt. ISME 14, 2936–2950. doi: 10.1038/s41396-020-0720-5, PMID: 32681158 PMC7784920

[ref146] Zeng D. D. Ye W. W. Xu M. Lu C. C. Tian Q. (2017). Rapid diagnosis of soybean root rot caused by *Fusarium culmorum* using a loop-Mediat ed isothermal amplification assay. J. Phytopathol. 165, 249–256. doi: 10.1111/jph.12556

[ref147] Zetsche B. Gootenberg J. S. Abudayyeh O. O. Slaymaker I. M. Makarova K. S. Essletzbichler P.. (2015). Cpf1 is a single RNA-guided endonuclease of a class 2 CRISPR-Cas system. Cell 163, 759–771. doi: 10.1016/j.cell.2015.09.038, PMID: 26422227 PMC4638220

[ref148] Zhang Y. Fang W. Yan D. Ji Y. Chen X. Guo A.. (2023). Comparison of drip-irrigated or injected allyl isothiocyanate against key soil-borne pathogens and weeds. Pest Manag. Sci. 79, 3860–3870. doi: 10.1002/ps.7590, PMID: 37256601

[ref149] Zhang H. Feng J. Manolii V. Strelkov S. E. Hwang S. F. (2015). Characterization of a gene identified in pathotype 5 of the clubroot pathogen *Plasmodiaphora brassicae*. Phytopathology 105, 764–770. doi: 10.1094/PHYTO-10-14-0270-R, PMID: 25689519

[ref150] Zhang X. Li F. Wang H. Wang F. (2023). Identification of tomato fusarium crown and root rot pathogen in Shandong province. J. Qingdao Agricult. Univ. 40, 24–29. doi: 10.3969/J.ISSN.1674-148X (In Chinese)

[ref151] Zhang J. X. Ling J. Xie B. Y. Yang Y. H. (2014). Rapid detection and identification of *Fusarium oxysporum* f.sp. *conglutinans* race 1 and race 2. Acta Phytopathol. Sin. 44, 586–594. doi: 10.13926/j.cnki.apps.2014.06.004

[ref152] Zhao T. Feng J. Sechler A. Randhawa P. Li J. Schaad N. W. (2009). An improved assay for detection of *Acidovorax citrulli* in watermelon and melon seed. Seed Sci. Technol. 37, 337–349. doi: 10.15258/sst.2009.37.2.08

[ref153] Zheng J. Wang X. L. Li Q. Yuan S. Wei S. Q. Tian X. Y.. (2018). Characterization of five molecular markers for pathotype identification of the clubroot pathogen *Plasmodiophora brassicae*. Phytopathology 108, 1486–1492. doi: 10.1094/PHYTO-11-17-0362-R, PMID: 29996697

[ref154] Zhong X. Yang Y. Zhao J. Gong B. B. Li J. R. Wu X. L.. (2022). Detection and quantification of *Fusarium oxysporum* f. sp. *niveum* race 1 in plants and soil by real-time PCR. Plant Pathol. J. 38, 229–238. doi: 10.5423/PPJ.OA.03.2022.0039, PMID: 35678056 PMC9343908

[ref155] Zhou X. Wang J. T. Liu F. Liang J. M. Zhao P. Tsui C. K. M.. (2022). Cross-kingdom synthetic microbiota supports tomato suppression of Fusarium wilt disease. Nat. Commun. 13:7890. doi: 10.1038/s41467-022-35452-6, PMID: 36550095 PMC9780251

[ref156] Zhu Z. X. Zheng L. Hsiang T. Yang G. L. Zhao D. L. Lv B.. (2016). Detection and quantification of *Fusarium commune* in host tissue and infested soil using real-time PCR. Plant Pathol. 65, 218–226. doi: 10.1111/ppa.12412

[ref157] Zou F. He L. G. Li X. M. Huang R. R. Ma H. G. (2023). Triplex PCR detection of three soil borne pathogens *Ralstonia solanacearum*, *Verticillium dahliae* and *Sclerotium rolfsii* in eggplants. J. Plant Prot. 50, 206–213. doi: 10.13802/j.cnki.zwbhxb.2023.2021044 (In Chinese)

